# Noise-enhanced stability in synchronized systems

**DOI:** 10.1126/sciadv.adx1338

**Published:** 2025-08-01

**Authors:** Zhan Shi, Qiangfeng Lv, Mengqi Fu, Xuefeng Wang, Zhilong Huang, Xueyong Wei, Marco Amabili, Ronghua Huan

**Affiliations:** ^1^ Department of Mechanics, Key Laboratory of Soft Machines and Smart Devices of Zhejiang Province, Zhejiang University, Hangzhou 310027, China.; ^2^ School of Engineering, Westlake University, Hangzhou 310030, China.; ^3^ Department of Physics, University of Konstanz, Konstanz 78457, Germany.; ^4^ Department of Engineering Mechanics, MIIT Key Laboratory of Dynamics and Control of Complex Systems, Northwestern Polytechnical University, Xi'an 710072, China.; ^5^ State Key Laboratory for Manufacturing Systems Engineering, Xi'an Jiaotong University, Xi'an 710049, China.; ^6^ School of Instrument Science and Technology, Xi'an Jiaotong University, Xi'an 710049, China.; ^7^ Department of Mechanical Engineering, McGill University, Montreal H3A 0C3, Canada.

## Abstract

Synchronization underpins coherence in natural and engineered systems, unifying dynamics and countering noise while remaining vulnerable to disturbances threatening the stability and risking desynchronization. Here, we present a counterintuitive approach: harnessing noise to dilute the energy of unwanted fluctuations from external disturbances, thereby enhancing stability while preserving synchronization through the system’s inherent noise suppression at the synchronized frequency. Through experiments with micromechanical oscillators and macroscale rotors, combined with stochastic averaging analysis, we show that this noise dilution effect improves synchronization efficiency, bolsters resistance to interference, and enhances long-term frequency stability. These findings position white noise of appropriate intensity as a dilution element for mitigating unwanted disturbances, providing previously unidentified insights into stability and resilience in complex synchronized systems.

## Teaser

White noise of appropriate intensity enhances the stability of synchronized systems by diluting disturbances.

## INTRODUCTION

Synchronization is profound in natural and engineered systems, promoting coherence across biological (*1*–*7*), physical (*8*–*13*), and engineering domains (*14*–*19*). It offers several key advantages: enhanced stability (*20*–*22*), improved efficiency (*23*, *24*), and noise mitigation (*25*–*30*), with scales spanning from quantum systems (*31*–*33*) to large-scale power grids (*34*, *35*). This broad applicability arises from the ability of weak interactions to align individual components’ phases and frequencies without overpowering their intrinsic dynamics, even in a noisy environment, preserving natural behavior while fostering collective order. However, such flexibility in phase adaptation is vulnerable to strong external disturbances, which lead to desynchronization, prolong recovery times, and degrade performance across diverse platforms (*36*–*40*). Here, we define disturbances as external influences that compromise the stability of synchronized systems—for example, wind turbulence destabilizing synchronized airplane engines (*41*), and intense signal interference disrupting synchronized microelectromechanical system (MEMS) oscillators (*42*, *43*).

Ensuring resilience against severe disturbances is therefore crucial for robust synchronization. To date, strategies such as increased coupling strength, nonlinear feedback control, and network-topology optimization have demonstrably improved stability (*44*, *45*), but they typically demand extra components—high-precision sensors, complex control circuitry, or continuous real-time monitoring—which adds substantial complexity and cost. Alternative approaches that achieve robust frequency stability and ease of integration (*46*–*48*)—for example, exploiting mode coupling—have shown promise in high-precision resonators, yet they remain challenging to implement in fully synchronized networks. In this work, we demonstrate that a controlled level of white noise—typically regarded as a disruptive influence—enhances the stability of synchronized systems across both micro- and macroscales implementations under external disturbances. We use the term “dilution effect” throughout the text to describe the phenomenon where noise dilutes adverse response induced by disturbances. Specifically, the two studied synchronized systems—electrostatically coupled microscale oscillators and mechanically coupled macroscale rotors—reveal that stochastic noise introduced at controlled levels enhances synchronization efficiency, improves frequency stability, and accelerates recovery from disturbances. Theoretical analysis based on stochastic averaging and numerical simulations shows that proper level of noise exerts a dilution effect on unwanted disturbances on synchronized systems and improved synchronization efficiency. Similar benefits of noise have been observed in unsynchronized systems, where it simplifies pattern recognition tasks (*49*), aids quantum data distillation (*50*), and improves collective behaviors such as fish schooling (*51*–*53*), suggesting that noise enhances system stability under certain conditions. Notably, in synchronized systems, the adverse effects of increased noise, such as reduced frequency stability, are mitigated through mechanisms like phase locking and collective oscillation, allowing the beneficial impacts of noise to dominate while minimizing its detrimental effects. This work highlights noise as a powerful tool for enhancing synchronization stability, with possible broad applications in science and engineering.

## RESULTS

### Principal concepts

**Fig. 1.**
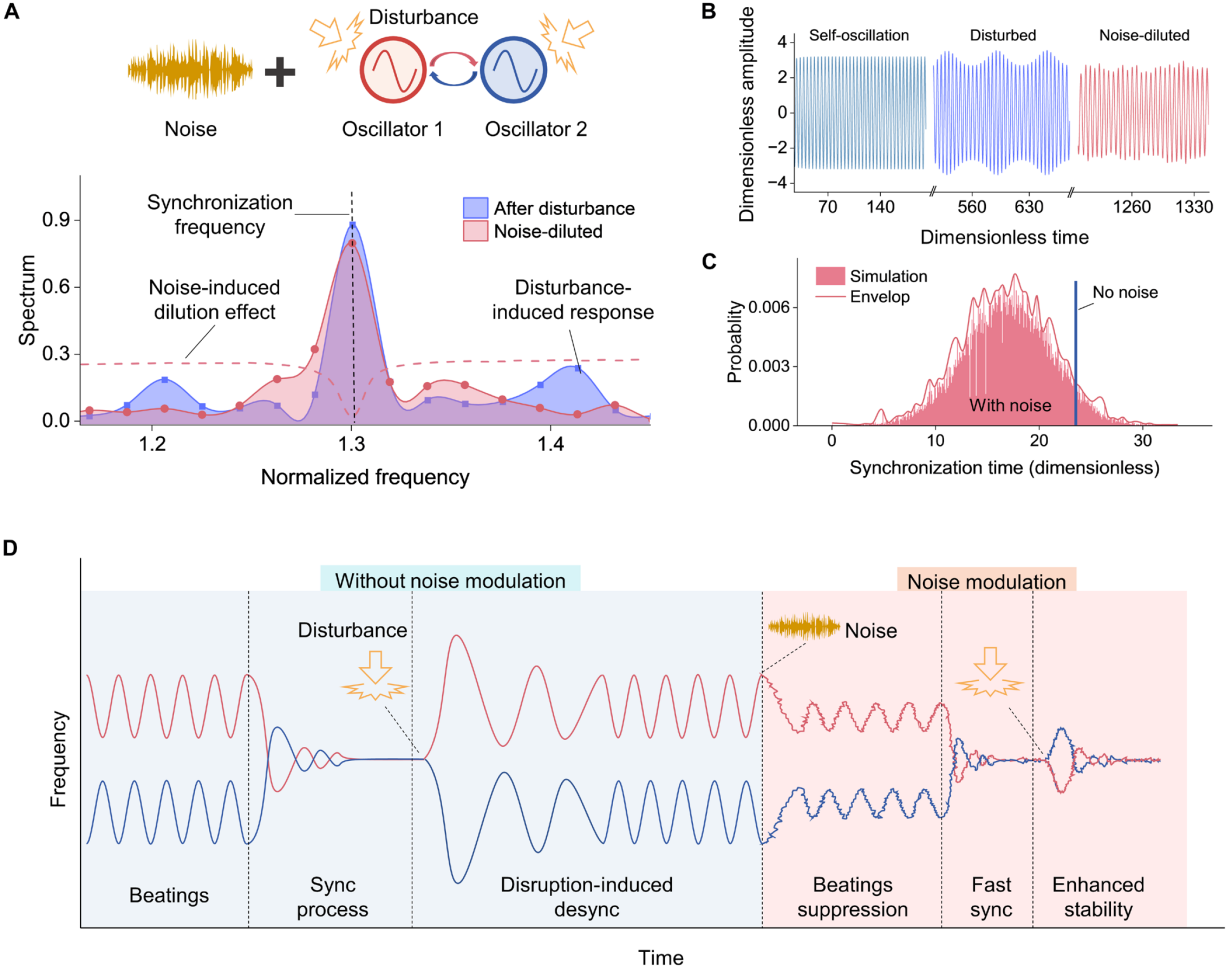
Concepts of the noise’s dilution effects on synchronized systems. (**A**) The simplified physical model of the synchronization of the coupled oscillators subjected to white noise. The bottom panel shows the noise dilution effects (pink dashed curve) on the external disturbance-induced response in the frequency domain, while the dilution effect is squeezed at the synchronization frequency. (**B**) Simulation of the time-domain amplitudes (dimensionless) of the synchronized systems in different stages: self-oscillation, beating (amplitude modulated) response under disturbance, and resynchronization under disturbance and noise. (**C**) Simulation of the synchronization time (dimensionless) with noise dilution effects, where the blue line is the simulated synchronization time without noise, and the pink area shows the synchronization time with the noise’s dilution. (**D**) Illustration of the coupled oscillators’ frequency response, which contains multiple stages: beating frequency stage, synchronization process stage, disturbance-induced desynchronization stage, noise modulation-induced acceleration of the synchronization process stage, and noise modulation enhanced stability stage, which can resist the same level of the disturbance.

Despite the complexity interaction of synchronization in various platforms, most of these systems can be effectively modeled as two coupled oscillators (*54*), as shown in Fig. 1A. Incorporating noise modulation in these systems, the generalized governing equations for the oscillators’ motion are

$$\begin{array}{c} \begin{array}{l} {\ddot{X}}_{1}+\eta_{1}{\dot{X}}_{1}+\omega_{01}^{2}X_{1}+\alpha_{1}X_{1}^{2}+\beta_{1}X_{1}^{3}=E_{1}\text{cos}\Phi_{1}\left(t\right)-G_{1}\left(X_{1}-X_{2}\right)+\xi_{1}\left(t\right)+X_{1}\xi_{2}\left(t\right),\\ {\ddot{X}}_{2}+\eta_{2}{\dot{X}}_{2}+\omega_{02}^{2}X_{2}+\alpha_{2}X_{2}^{2}+\beta_{2}X_{2}^{3}=E_{2}\text{cos}\Phi_{2}\left(t\right)-G_{2}\left(X_{2}-X_{1}\right)+\xi_{1}\left(t\right)+X_{2}\xi_{2}\left(t\right) \end{array} \end{array}$$ (1)

where $\eta_{i}\left(i=1,2\right)$ are damping coefficients, and $\omega_{0i}$ are natural frequencies of two oscillators. The coefficients $\alpha_{i}$ and $\beta_{i}$ represent quadratic and cubic nonlinearities, respectively. $E_{i}$ denote the feedback forces from closed-loop control responsible for sustaining oscillations, and Φ*_i_*(*t*) are instantaneous phases of the two oscillators. $G_{i}$ represent linear interaction between oscillators, which dominates in parallel plate electrostatic force coupling structures (*55*). The terms $\xi_{i}\left(t\right)$ denote independent white Gaussian noises with correlation functions $E\left[\xi_{i}\left(t\right)\xi_{i}\left(t+\tau \right)\right]=2D\delta \left(\tau \right)$ , where *D* is the noise intensity. In general, additive noise arises from mechanical thermal fluctuations, while multiplicative noise is associated with fluctuations in the driving and detection signals (*56*).

The flexible nature of phase in synchronized systems makes it easy for external disturbances to affect the synchronized oscillator frequency, causing frequency mismatches. When two oscillators vibrate at slightly different but closely spaced frequencies, the resulting motion presents a beating phenomenon associated with amplitude modulation (*57*). This effect can induce phase drift, leading to partial or complete desynchronization, thereby compromising system stability. Noise in oscillators typically induces a dilution effect, broadening spectral lines at harmonic frequencies and degrading frequency precision, as illustrated by the simulation results in fig. S1A. However, when noise is strategically introduced into the synchronized oscillator, it can disperse energy that would otherwise concentrate in beat frequency responses, thereby mitigating the impact of external disturbances. In our simulations, disturbances were introduced as sudden 30% increases in amplitude and frequency to simulate a rapid energy injection into the system (e.g., an impulse or resonant excitation). Notably, the noise-mitigation effects of synchronization effectively suppress random fluctuations at synchronization frequency, preserving synchronization performance (Fig. 1A and fig. S1B).

The behavior is further demonstrated in Fig. 1B; the noise-induced dilution effects effectively reduce amplitude fluctuations in the beat frequency stage, enabling the system to synchronize more quickly and robustly against external disturbances (Fig. 1C), where we performed 500 simulations of synchronization time between two oscillators with a fixed initial frequency detuning ( $\omega_{1}-\omega_{2}=0.1$ ) under two conditions: with noise modulation D=0.001 (pink) and without it (blue). The enhanced synchronization efficiency is further demonstrated in fig. S2, where we numerically simulated two oscillators’ time evolution frequencies ( $\omega_{1}$ and $\omega_{2}$ ) and phase difference ( Δϕ ) based on eq. S13 under different noise intensities applied equally to both oscillators ( $D_{1}=D_{2}=D$ , D=0 , $1\times 10^{-3}$ , $2\times 10^{-3}$ , $3\times 10^{-3}$ , and $1\times 10^{-2}$ , respectively). The synchronization time first decreases, then increases, and lastly becomes unable to synchronize due to excessive level of noise. The statistical analysis reveals that white noise of appropriate intensity effectively suppresses the disturbance-induced amplitude fluctuation and accelerates synchronization, demonstrating its potential to enhance synchronization efficiency and resilience.

The noise-induced dilution effect offers multiple benefits as a modulation strategy. During synchronization, noise reduces beat frequencies and enhances synchronization efficiency. Moreover, as synchronized oscillators encounter disturbances, noise mitigates their impact, making the system more robust to external disturbances, as demonstrated in Fig. 1D. To prove the assumptions, we carried out experiments on two distinct systems: two electrostatically coupled micromechanical oscillators and two mechanically coupled macroscale rotors.

### Micromechanical oscillators

#### Device characteristics and synchronization characterization

**Fig. 2.**
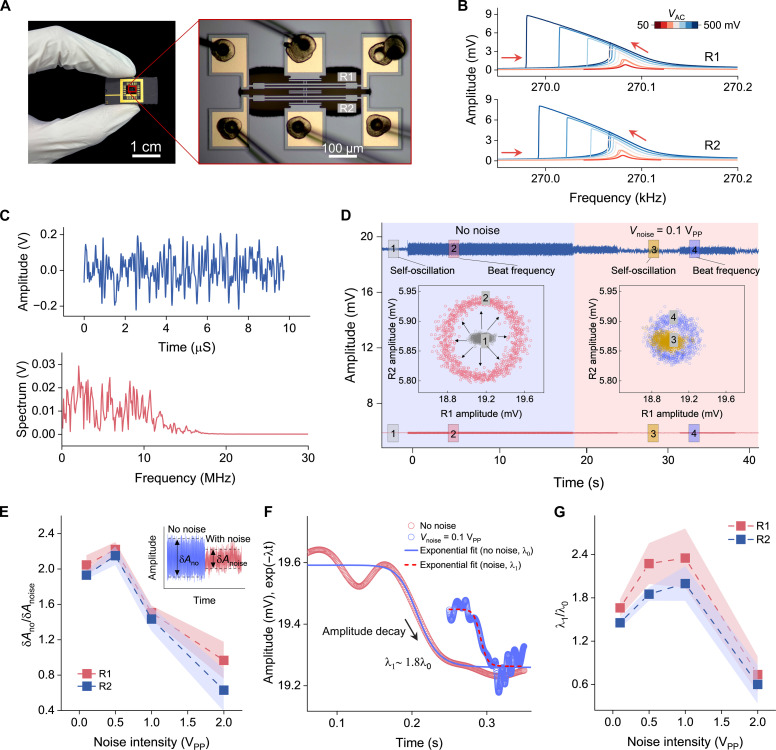
Device characteristics and noise effects in micromechanical oscillators. (**A**) Optical image of the micromechanical straight-beam resonators, with the zoomed-in view highlighting the resonators (R1 and R2). (**B**) Measured amplitude-frequency responses of R1 and R2 under varying excitation amplitudes (50 to 500 mV), showing softening nonlinear behavior with increasing drive levels. (**C**) Time-domain response and frequency spectrum of the noise. The noise has a frequency bandwidth of 10 MHz and a voltage of 0.1 V_PP_. (**D**) Time-domain amplitudes of R1 (blue) and R2 (red) without noise modulation (blue-shaded region) and with noise modulation (pink-shaded region). Insets depict the amplitude relationships between the two oscillators: the left inset shows the relationship before (gray) and after (pink) a disturbance without noise, while the right inset shows the relationship before (yellow) and after (blue) a disturbance with white noise (0.1 V_PP_). In these cases, *V*_DC_ = 20 V_PP_, *V*_AC_ = 300 mV. (**E**) Amplitude fluctuation ratio (δ*A*_1_/δ*A*_2_) of R1 at disturbed state without noise (δ*A*_1_) and with noise (δ*A*_2_) at different noise levels (0.1, 0.5, 1, and 2 V_PP_). (**F**) Amplitude decay of R1 with and without noise modulation. Experimental data (dots) are fitted with exponential decay curves exp(−λt) (solid lines), where the fitted $\lambda_{0}=20$ , $\lambda_{1}=36$ , demonstrating enhanced decay rate with noise. (**G**) Noise effects on both oscillators’ amplitude decay. The amplitude decaying ratio between without noise and with noise with varying noise intensity (0.1, 0.5, 1, and 2 V_PP_, respectively). The DC voltage in all experiments was set to *V*_DC_ = 20 V_PP_.

The micromechanical system under study consists of two electrostatically coupled straight beam resonators (R1 and R2), as depicted in Fig. 2A. Both beams are fabricated from single-crystal silicon using a silicon-on-insulator (SOI) process, with identical dimensions of 500 μm by 10 μm by 25 μm. Each beam is clamped at both ends and anchored with gold electrodes, which serve dual purposes: detecting vibrations via the piezoresistive properties of silicon and injecting electrothermal current ( $I_{\text{th}}$ ) to tune natural frequencies of the resonators. The outer middle plates of both beams act as driving elements. The beams are excited electrostatically using a combination of direct current (DC) voltage, *V*_DC_, and alternating current (AC) voltage, $V_{\text{AC}}\text{cos}\left(\Omega t\right)$ , with Ω and *t* representing the excitation angular frequency and time, respectively. The detected vibration signal first passes through a differential amplification module to minimize parasitic feedthrough, then through the built-in filter of the Lock-in Amplifier (Zurich Instruments, HF2LI) to obtain a pure vibration signal (see fig. S3 for details of the experimental setup).

To address minor geometric imperfections arising from fabrication, we varied *I*_th_ from 0.4 to 5.6 mA of both resonators, respectively, to tune their natural frequencies, thereby achieving an approximately identical operating frequency region marked as the synchronizable range (see fig. S4 for details). To further investigate the resonance frequency characteristics within this synchronizable range, the electrothermal currents were set at 3 mA for R1 and 5 mA for R2, resulting in measured natural frequencies of 269,371 and 269,394 kHz, respectively, with an initial frequency detuning of 23 Hz. We then swept the excitation frequency around each resonator’s natural frequency at different AC excitation levels (*V*_AC_ = 50 to 500 mV, *V*_DC_ = 20 V_PP_; where V_PP_ denotes peak-to-peak voltage) to investigate their resonance properties. The amplitude-frequency response curves for both resonators, shown in Fig. 2B, exhibit typical softening nonlinear behavior at higher driving magnitudes. The resonator parameters were extracted from these response curves and used in the following calculations (see section S5 and table S1 for detailed parameter characterization).

To enable synchronization, self-sustained oscillation is established through a built-in phase-locked loop (PLL), as depicted in fig. S5A. The linear and nonlinear periodic self-oscillation states are validated through phase-space analysis, represented by the oscillation displacement and velocity $\left(x,\dot{x}\right)$ (figs. S5B to S5G). Furthermore, the phase locking point remains stable under varying nonlinear conditions, ensuring robust control of oscillations and enabling systematic experimental characterization (fig. S6). We conducted experiments to explore the synchronization range without and with a “white noise” of appropriate intensity and bandwidth. In the experiments, we aimed to use white noise; however, achieving ideal white noise was not feasible. Instead, we used ultra-wideband noise with a 10-MHz bandwidth as an approximation, with its amplitude-time and amplitude-frequency responses shown in Fig. 2C. Given that the oscillators’ oscillation frequencies are both approximately 270 kHz, the 10-MHz noise is substantially broader in comparison, allowing it to effectively serve as white noise in our system. The PLL facilitates tunability of the oscillation frequencies of both oscillators via phase delay control, enabling detailed characterization of the synchronization region. The relationship between self-oscillation frequency ( $\Omega_{0}$ ) and phase delay ( $\phi_{0}$ ) of the feedback force ( $f_{0}$ ) is given by $\Omega_{0}\approx \frac{1}{\sqrt{2}}{\left\{1+{\left[1+3\beta Q^{2}f_{0}^{2}{\text{sin}}^{2}\left(\phi_{0}\right)\right]}^{\frac{1}{2}}\right\}}^{\frac{1}{2}}$ (see derivation in the Supplementary Materials), where β and Q represent the equivalent nonlinearity and quality factor, respectively (*58*). In our experiments, we measured the oscillation frequencies of both oscillators. Figure S7A depicts the relationship between oscillation frequency and phase delay, with the inset showing real-time modulation of the oscillation frequency as the phase delay is varied.

Measurement of the synchronization region, both without and with noise, allows for further characterization of synchronization time. The synchronization region between two oscillators was measured and shown in fig. S7B, which depicts the real-time synchronization process. After achieving synchronization, we adjusted the phase delay to modify R1’s oscillation frequency. Because of the frequency entrainment effect in the synchronized state, R2’s frequency shifted correspondingly. However, when synchronized frequency exceeded the upper threshold, the oscillators desynchronized, reverting to their individual self-oscillation frequencies. The region where the oscillators maintained identical frequencies is highlighted in pink, representing the measured synchronization range of 29.1 Hz.

We then investigated the synchronization bandwidth under influence of noise. Figure S7 (C and D) shows the measured synchronization ranges with white noise up to a frequency of 10 MHz and amplitudes of 0.1 and 1 V_PP_, respectively. The synchronization bandwidths were measured to be 31.3 and 29.5 Hz, indicating that the added noise has an almost negligible effect on the synchronization bandwidth in our case. To ensure synchronization in all subsequent experiments, both oscillators’ frequencies were conducted within the pink-highlighted region.

#### Noise-induced dilution effects

We first verified noise-induced dilution effects on stabilizing the two oscillators by demonstrating the disturbance-induced amplitude fluctuation suppression. In our experiments, disturbances are introduced by injecting an external signal with a frequency Ω_p_ close to, but slightly different from, the synchronization frequency Ω_s_ into two oscillators simultaneously. This frequency mismatch (ΔΩ = Ω_p_ − Ω_s_) leads to a beating phenomenon between two oscillators, effectively simulating a disturbed state (see detailed characterization of the effects of external signal amplitude and frequency detuning on amplitude fluctuations in both oscillators in fig. S8). As illustrated in Fig. 2D, when two oscillators are synchronized, the relationship between their amplitudes appears as discrete dots (state 1, gray dots), indicating relatively small amplitude fluctuations (δ*A*_1_ < 0.2 mV, δ*A*_2_ < 0.012 mV). To explore noise effects on the synchronized system, we examined the beating stage, where an external signal (30 mV, ΔΩ = 40 Hz) with a frequency near the oscillation frequency was applied; this beating phenomenon induced relatively large amplitude fluctuations (δ*A*_1_ > 1 mV, δ*A*_2_ > 0.13 mV), inducing beating and forming larger external circles (state 2, pink dots). When white noise up to 10 MHz (0.1 V_PP_) is applied, the previously undisturbed state becomes slightly noisy (state 3, yellow dots, δ*A*_1_ ~ 0.36 mV, δ*A*_2_ ~ 0.024 mV). When the same level disturbance signal is reintroduced, both oscillators’ amplitudes showed notably smaller fluctuations (δ*A*_1_ < 0.5 mV, δ*A*_2_ < 0.07 mV) compared with the no noise dilution effect condition, making the disturbed state (state 4, blue dots) remaining closer to the undisturbed state compared to the scenario without noise. We further characterized the noise dilution effect by varying the noise intensities from 0 to 2 V_PP_, and quantified its impact by calculating the ratio of amplitude fluctuations without and with noise (Fig. 2E). The results show that the introduced noise effectively suppresses amplitude fluctuations at specific intensities (0.1, 0.5, and 1 V_PP_). As the noise intensity reaches 2 V_PP_, the amplitude fluctuations of both oscillators become more pronounced than in stage 2.

Further, we showed that the introduced noise increases the amplitude decay rate during recovery from the beating response stage to the self-oscillation stage, as depicted in Fig. 2F. After removing the disturbing signal (30 mV, ΔΩ = 40 Hz), the amplitudes of both oscillators decayed to stable values in both the absence and presence of noise. The decay rate with noise (λ_1_), extracted from exponential fitting, was 1.8 times greater than that without noise (λ_0_). We further quantified the ratio λ_1_/λ_0_ across noise intensities of 0.1, 0.5, 1, and 2 V_PP_ (Fig. 2G). For 0.1 to 1 V_PP_, λ_1_/λ_2_ > 1, indicating that noise accelerates recovery from the perturbed state. However, at 2 V_PP_, the decay slowed due to large amplitude fluctuations induced by excessive noise. These two effects of noise on synchronized oscillators—the suppression of disturbed amplitude fluctuations and the acceleration of amplitude decay toward the stable state—enable further characterization of the noise effects on the synchronization.

**Fig. 3.**
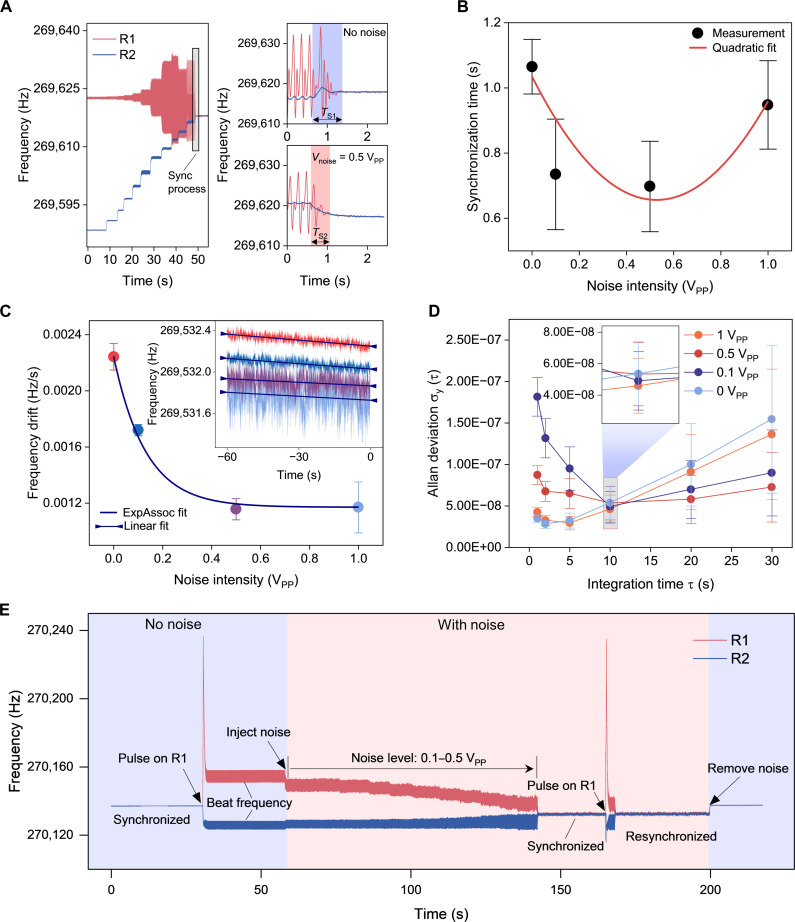
Device characteristics and experimental setup. (**A**) Time domain of the synchronization process of two coupled oscillators working in linear (blue) and nonlinear (red) regimes, respectively. Insets show the detailed synchronization process of two oscillators without and with noise modulation, where the noise intensity is 0.5 V_PP_. The shaded color area indicates the synchronization time without noise (blue) and with noise (pink). (**B**) Measured synchronization time at different noise intensities. (**C**) Frequency drift of the synchronized oscillators at a time range of 1 min under different noise intensities. Inset is the real-time frequency of the synchronized oscillators. (**D**) Measured Allan deviation at noise intensities 0 to 1 V_PP_. (**E**) Experimental demonstration of the frequency response of coupled oscillators reveals multiple dynamic stages: initial synchronized, pulse-induced desynchronization, emergence of a beating-frequency regime, noise-enhanced resynchronization, and a noise-enhanced stability that withstands equivalent levels of disturbance. Notably, the improved frequency stability persists even after the noise is removed.

We then investigated the effect of noise on synchronization time under three different conditions: linear (i.e., small amplitude) oscillator synchronizing with linear oscillator (L-L), linear oscillator synchronizing with nonlinear (large amplitude) oscillator (L-N), and nonlinear oscillator synchronizing with nonlinear oscillator (N-N). Details of the measurements are provided in fig. S9. Here, we illustrate the L-N case in Fig. 3A, where R1 and R2 operate in linear and nonlinear states, respectively. As R2’s frequency is tuned to approach R1’s frequency using phase control, R1’s oscillation frequency shifts accordingly. However, because R1 operates in the linear regime, its frequency cannot be notably adjusted, resulting in a typical beat frequency pattern, with a frequency fluctuation (δω_1_) of ~22 Hz. After fine-tuning R2’s frequency, we identified the final point where the two oscillators synchronize. When R2’s phase is tuned to this point, both oscillators’ frequencies rapidly coordinate, moving the system into a synchronized state with a synchronization time (*T*_S1_) of ~0.9 s. Insets present the detailed synchronization process without and with noise modulation. The injected noise with an intensity of 0.5 V_PP_ clearly decreases the beat frequency fluctuations (δω_2_ ~ 15 Hz), demonstrating the dilution effect, facilitating quicker synchronization of the oscillators (*T*_S2_ ~ 0.5 s).

We then conducted experiments on characterizing noise intensity effects on synchronization time between the oscillators (see identification details of synchronization time in fig. S10), as shown in Fig. 3B. As noise intensity increases from 0.1 to 0.5 V_PP_, the synchronization time initially decreases, attributable to the noise dilution effect on the energy of frequencies near the synchronized frequency during the beat frequency stage. However, once the noise intensity reaches 1 V_PP_, synchronization time increases again. This behavior occurs because excessive noise makes the oscillators too unstable to achieve synchronization effectively, leading to a marked longer measured synchronization time. This variation in synchronization time with noise intensity qualitatively matches the simulation results presented in fig. S2.

Synchronization can improve frequency stability, which is important in timekeeping applications, while noise generally degrades it. Counterintuitively in our experiments, as noise intensity increased from 0 to 1 V_PP_, the noise fluctuations became more pronounced, yet the frequency drift measured over 1 min decreased from 0.023 to 0.012 Hz/s, a reduction of around 50%, as shown in Fig. 3C. To quantitatively assess frequency stability, we used the widely adopted Allan deviation (*59*), calculated as $\sigma_{y}\left(\tau \right)=\sqrt{\frac{1}{2\left(N-1\right)}\sum_{i=1}^{N-1}{\left({\bar{y}}_{i+1}^{\tau }-{\bar{y}}_{i}^{\tau }\right)}^{2}}$ , to characterize frequency fluctuations. ${\bar{y}}_{i}^{\tau }$ denotes the average fractional frequency measured over the *i*th time window of length τ, and ${\bar{y}}_{i+1}^{\tau }$ is the average over the immediately next window of the same duration. A smaller Allan deviation corresponds to higher frequency stability. Our measurements (Fig. 3D) show that while the noise (10 MHz, 0.1 to 1 V_PP_) adversely affects short-term (<10 s) frequency stability, it can enhance frequency stability at a long timescale (>10 s). The improvement of the frequency stability on a long timescale is beneficial in applications like timekeeping, and could be attributed to the noise-induced dilution effects on external disturbances in experimental conditions.

We then demonstrated that noise enhances the stability of synchronized oscillators under strong disturbances (Fig. 3E). Initially, the two oscillators were synchronized. A strong pulse signal (0.5 mA peak current, 50 ms duration) was injected into R1, inducing a ~20-Hz frequency shift via the electrothermal effect and causing desynchronization. The resulting frequency mismatch (~28 Hz) led to a persistent beat phenomenon, preventing resynchronization. However, when noise was introduced to both oscillators, the beat amplitude diminished, and at a noise intensity of 0.5 V_PP_, the oscillators resynchronized. Upon reapplying the same pulse signal, the oscillators again desynchronized, but now with reduced frequency mismatch (~9 Hz) at the beat stage, and were able to autonomously resynchronize without further modulation. Although the synchronized frequency appeared noisy during this process, it became stable quickly once the added noise was removed, highlighting the utility of noise dilution in maintaining robust synchronization.

### Macroscale rotors

#### Experimental setup and rotor characteristics

**Fig. 4.**
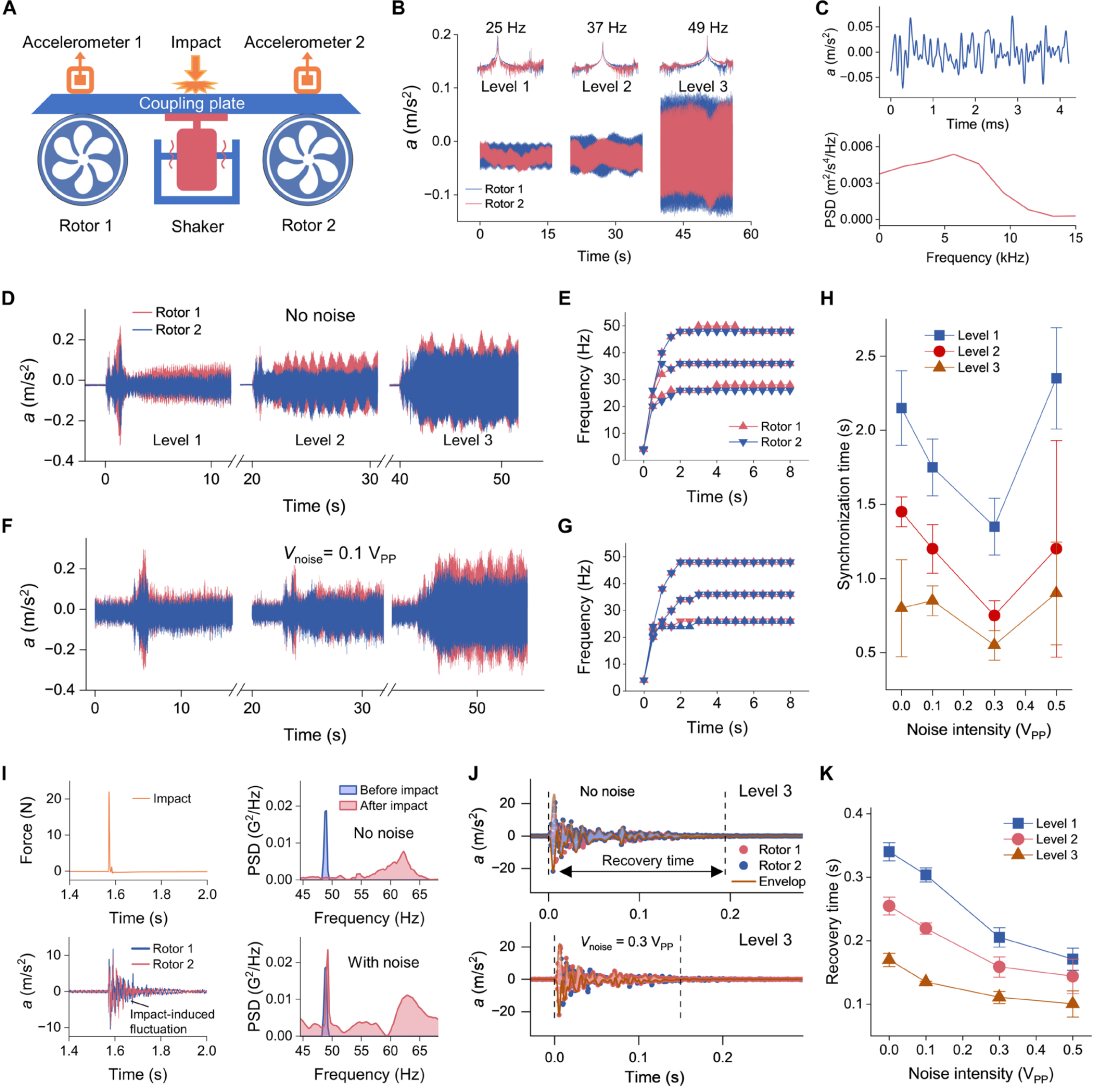
Experimental scheme and results for noise effects on coupled macroscale rotors. (**A**) Experimental setup: Two rotors are mounted on the wings of an airplane-shaped aluminum alloy plate; accelerometers are affixed atop each rotor. Broadband noise is introduced via a shaker; mechanical impacts are applied at the center of the plate. (**B**) Time-domain acceleration signals and corresponding frequency spectra at three drive levels (level 1 to 3), with red and blue traces representing rotors 1 and 2. (**C**) Time and frequency domain characterization of the input noise (0.1 V_PP_, limited to 10 kHz). (**D** and **E**) Synchronization dynamics and real-time frequency evolution during rotor startup without noise. (**F** and **G**) Same as (D) and (E), with noise (0.1 V_PP_, limited to 10 kHz). (**H**) Synchronization time without noise and with noise intensities of 0.1, 0.3, and 0.5 V_PP_. (**I**) Impact effects on rotors without and with noise. Top left: Time-domain impact signal generated by an instrumented hammer. Bottom left: Rotor acceleration response following the impact. Top and bottom right, power spectral densities (PSDs) before and after impact without and with noise, respectively. (**J**) Time-domain acceleration traces at level 3, without and with 0.3 V_PP_ noise, following an impact disturbance. Recovery time is defined as the duration required for the perturbed signals to return to a stable amplitude, quantified using the envelope of the acceleration traces. (**K**) Recovery time under identical impact conditions without noise and across varying noise intensities (0.1, 0.3, and 0.5 V_PP_).

Beating oscillations between synchronized engines, often induced by turbulence in the troposphere, can destabilize aircraft structures, degrade passenger comfort, and accelerate structural fatigue. To emulate this scenario and probe strategies for suppressing such instabilities, we constructed a tabletop platform comprising two rotors mounted at either end of a flexible aluminum plate. This approach aims to offer insights into real engine behavior by accelerating synchronization and reducing fuselage vibrations. The experimental setup, shown in Fig. 4A, consists of two rotors (rotor 1 and rotor 2, which are electric motors) mounted at each end of a plane-shaped aviation aluminum alloy plate (see fig. S11 for a photo of the experimental setup). This configuration allows for remote control of each rotor at three different speeds (1500, 2220, and 2940 rpm, corresponding to 25, 37, and 49 Hz), with the plate serving as an energy transfer medium between the rotors while also receiving noise excitation from a connected electrodynamic actuator (vibrating platform). Vibration signals from the coupling plate, which supports the rotors, are captured using accelerometers; the instrumentation is detailed in Materials and Methods.

We first characterized the resonance properties of the rotors operating independently, measuring their stable vibration at different levels with accelerometers. The time-domain acceleration responses, shown in Fig. 4B, indicate stronger vibration amplitudes at higher speeds and frequencies. Using the Fast Fourier transformation, we identified the primary response frequencies at the location of rotor 1 as 25, 37, and 49 Hz at speed levels 1, 2, and 3, respectively, with rotor 2 closely matching these frequencies, facilitating easy synchronization.

#### Characterization of the noise effects on synchronization

Next, we measured the synchronization time between two rotors without noise perturbation. We measured the real-time accelerations of two rotors during experiments, then calculated the real-time frequency. The time-domain response and real-time frequency measurements are, respectively, shown in Fig. 4 (D and E), revealing a synchronization process similar to that of micromechanical oscillators. Initially, the two rotors exhibit distinct vibration frequencies, then their frequencies gradually converge, achieving synchronization after approximately 2 s.

We then characterized the synchronization process with noise introduced to the rotors. Here, we applied noise with a frequency of 10 kHz (Fig. 4C), which falls within the shaker’s operating range and is notably higher than the rotor’s various oscillation frequencies (<50 Hz). As shown in Fig. 4 (F and G), after the noise is introduced, the synchronization process is accelerated and remains more stable compared to the case without the noise. The measured synchronization time exhibits a clear nonmonotonic dependence on noise intensity across all three drive levels (Fig. 4H). At level 1, the synchronization time initially decreases from ~2.2 s at zero noise to a minimum of ~1.3 s at a noise intensity of 0.3 V_PP_, before rising again to ~2.3 s at 0.5 V_PP_. A similar trend is observed at level 2, where the synchronization time drops from ~1.5 s to a minimum of ~0.8 s, then rises to ~1.2 s. At the highest drive level (level 3), the system exhibits the fastest overall synchronization, with times ranging from ~0.8 s down to a minimum of ~0.6 s at 0.3 V_PP_, and rising again to ~0.9 s. This characteristic nonmonotonic behavior confirms that moderate noise facilitates faster synchronization. However, excessive noise eventually becomes disruptive to synchronization.

To highlight the beneficial effects of noise, we examined the vibration behavior of the coupling plate when subjected to an impact. In the experiments, an impact disturbance was introduced by dropping a 304 stainless steel sphere (radius: 2 cm; mass: 265 g) from a height of 30 cm onto a fixed point at the center of the coupling plate. This produced a peak acceleration of approximately 25 m/s^2^. To quantitatively assess the effect of this impact on the frequency shift of the rotors, we used an instrumented impact hammer (Brüel & Kjær, Type 8206) and calibrated it to replicate the mechanical response induced by the falling sphere (fig. S12).

Figure 4I illustrates the effects of impulsive disturbances on the dynamics of coupled rotors, both in the absence and in the presence of noise. The time-domain impact signal (top left) represents a sharp, short-duration force applied by the instrumented hammer, which triggers transient oscillations in the rotor accelerations (bottom left). Without noise, the impact introduces a notable shift in the power spectral density (PSD), resulting in the emergence of a broad secondary spectral component beyond the synchronized frequency peak (top right). In contrast, when noise is present (bottom right), the PSD indicates a more uniform spectral broadening while keeping the synchronization frequency peak, suggesting that the noise suppresses the sharp spectral displacement caused by the impact. These results indicate that noise can play a stabilizing role by mitigating the spectral distortions induced by impulsive disturbances.

The impact signal induced a pronounced disturbance in the acceleration signals of both rotors, followed by a finite recovery period during which the system returned to a stable, synchronized state, quantified as the recovery time. As shown in Fig. 4J, the acceleration traces of two coupled rotors demonstrate the recovery dynamics following an impact, both in the absence and in the presence of injected noise. Without noise, the system exhibits slower recovery to synchronized steady state, whereas moderate noise (*V*_noise_ = 0.3 V_PP_) accelerates recovery, as indicated by a faster decay in the amplitude envelope and shorter recovery time. Quantitative analysis across varying noise intensities shows that recovery time decreases with increasing noise levels (Fig. 4K). These findings demonstrate that with the influence of an appropriate level of noise, the coupling plate can better resist disturbances.

## DISCUSSION

In this paper, we explore how the effect of noise can be transformed from a detrimental element into a beneficial one in synchronized systems. Our experiments, conducted with microscale electrostatically coupled micromechanical oscillators and macroscale mechanically coupled rotors, demonstrate that with an appropriate level of white noise intensity, coupled systems achieve faster synchronization, improved robustness against external disturbances, and sustained enhanced frequency stability over extended periods. We modeled the influence of noise using a simplified physical framework of two coupled oscillators subjected to stochastic disturbances. Analysis based on the stochastic averaging method suggests that moderate noise levels can accelerate synchronization, whereas excessive noise may have the opposite effect.

Despite these promising results, we recognize certain limitations. Practical challenges arise with the use of real white noise—simulated in our experiments using ultrawide bandwidth noise—and the piezoresistive effect for signal detection in the MEMS, which can introduce minor frequency shifts. Despite these limitations, our findings remain robust, as demonstrated by consistency across two validation standards, confirming the reliability of the results.

Looking ahead, future research could explore the effects of both narrow- and wide-band noise on synchronized systems, as well as their impact on more complex oscillator networks. Beyond fundamental physics, the utilization of white noise may serve as a strategy to enhance performance in several applications. For instance, in timing systems and frequency references, controlled noise could improve long-term stability without the need for additional circuitry. In communication systems, synchronization enhanced by noise could reduce phase jitter and improve signal coherence. In MEMSs and inertial sensing, where coupling multiple units can be challenging due to environmental noise, our findings suggest that controlled noise injection may stabilize the networked performance.

In terms of feasibility, our experimental validation across both microscale and macroscale systems, including the use of widely accessible white noise sources and standard signal detection methods, demonstrates that such noise-induced effects are not limited to idealized models but are realizable in practice. In addition, the generality of the coupling framework and noise implementation supports scalability to other physical domains, including neuromorphic computing, biological pacemakers, and synchronized robotics, where robust coordination is often required under fluctuating conditions.

In summary, this research underscores the constructive role of noise in synchronization, with broader implications for systems involving dynamic interactions between different modes or components, and with feasible paths toward implementation in both scientific and engineering applications.

## MATERIALS AND METHODS

### Fabrication of the micromechanical beams

The microresonators were fabricated using a commercial SOI multi-user MEMS processes micromachining process, beginning with a 25-μm SOI wafer. The fabrication proceeded through the following eight steps:

1) A phosphosilicate glass (PSG) layer was deposited, and phosphorus was driven into the top silicon surface. The PSG was then removed by wet chemical etching.

2) The photoresist was exposed through the first-level mask (pad metal layer) and developed. A metal stack was deposited over the patterned photoresist via e-beam evaporation.

3) The second-level mask (SOI) was used to expose and develop the photoresist, followed by deep reactive ion etching (DRIE) to etch the silicon down to the oxide layer.

4) Reactive ion etching removed the bottom-side oxide layer, and a subsequent wet oxide etch cleared the regions defined by the deep trench patterning mask.

5) The frontside protection was stripped via a dry etch, and any remaining exposed oxide was eliminated from the top surface using a vapor-phase HF process.

6) A separate silicon wafer was processed to create a shadow mask for metal patterning, with DRIE used to etch entirely through the mask wafer.

7) The shadow mask was aligned and temporarily bonded to the SOI wafer.

8) Upon removal of the shadow mask, a patterned metal layer remained on the SOI wafer.

### Experimental setup

#### Characterization of micromechanical resonators

For the microscale characterization of the micromechanical resonators, we use a power supply (Keysight E36312A) to provide the static DC voltage signal and a lock-in amplifier (Zurich Instruments HF2LI) to generate the alternating voltage signal for beam actuation. The detected vibration signal first passes through a differential amplifier (Texas Instruments OPA 656U) before being transmitted to the lock-in amplifier for processing. Noise is added using a waveform generator (Keysight 33600A).

#### Characterization of macroscale rotors

For the macroscale characterization of the two rotors (OPOLAR, MWF066, Brushless Motor, 94 mm by 240 mm by 278 mm, max 3000 r/min), we used a waveform generator (Keysight 33600A) to generate the noise signal, which was then amplified using a power amplifier (TIRA BAA500). This signal actuated an electrodynamic actuator (TIRA S51120) to drive the wing-shape plate (aluminum alloy). The resulting vibration was detected using piezoelectric accelerometers (PCB Piezotronics, 352C33) and processed with commercial software (Labgenius).

### Numerical simulations

Numerical calculations based on the theoretical model were performed using MATLAB (R2023b).

## Supplementary Materials

### This PDF file includes:

Sections S1 to S6 Figs. S1 to S12 Table S1 References
