# Supplementary material for: Noise-enhanced stability in synchronized systems
Source: Sci Adv. 2025 Aug 1;11(31):eadx1338. doi: 10.1126/sciadv.adx1338 (PMC12315954; doi:10.1126/sciadv.adx1338)
Supplement: Supplementary file 1 — Sections S1 to S6 Figs. S1 to S12 Table S1 References [file sciadv.adx1338_sm.pdf]

Science Advances
AAAS

Supplementary Materials for

Noise-enhanced stability in synchronized systems

Zhan Shi *et al.*

Corresponding author: Ronghua Huan, rhhuan@zju.edu.cn; Marco Amabili, marco.amabili@mcgill.ca; Xueyong Wei, seanwei@xjtu.edu.cn

Sci. Adv. **11**, eadx1338 (2025)
DOI: 10.1126/sciadv.adx1338

This PDF file includes:

Sections S1 to S6
Figs. S1 to S12
Table S1
References

Supplementary Text

S1. Theoretical modeling

We consider the following two coupled nonlinear oscillators of hardening type (i.e., with cubic nonlinearity) under stochastic excitations. The equations of motion are

$$\begin{aligned}\ddot{X}_1 + \eta_1\dot{X}_1 + \omega_{01}^2 X_1 + \alpha_1 X_1^2 + \beta_1 X_1^3 &= E_1\cos\Phi_1(t) - G_1\left(X_1 - X_2\right) + \xi_1(t) + X_1\xi_2(t),\\ \ddot{X}_2 + \eta_2\dot{X}_2 + \omega_{02}^2 X_2 + \alpha_2 X_2^2 + \beta_2 X_2^3 &= E_2\cos\Phi_2(t) - G_2\left(X_2 - X_1\right) + \xi_1(t) + X_2\xi_2(t),\end{aligned} \tag{S1}$$

where η_i are damping coefficients, and ω_{0i} are the natural frequencies of two resonators, α_i and β_i are the quadratic and cubic nonlinearity terms, respectively, which can be approximated as the effective cubic coefficient $\tilde{\beta}_i = \beta_i - \alpha_i / \omega_{0i}$ (*60*). E_i are the feedback forces in the closed-loop control for building the oscillation. G_i are the linear interaction between the oscillators, which is dominant in parallel plate electrostatic force coupled structures (*55*); $\xi_i(t)(i = 1,\ 2)$ are independent white Gaussian noise with correlation functions $E[\xi_i(t)\xi_i(t+\tau)] = 2D\delta(\tau)$, where *D* is the noise intensity.

Eq. (S1) is a typical system of two stochastic nonlinear differential equations. Considering the relatively weak nonlinear effect (where the softening effect only drifts the natural frequency of 0.56%), thus the 2-DOF equation can be solved by utilizing the stochastic averaging method. Under certain conditions (*61*), the solution of system (S1) can be expressed as the following:

$$\begin{aligned}&X_i(t) = A_i\cos\Phi_i(t), \quad \dot{X}_i(t) = -A_i\nu_i(A_i,\Phi_i)\sin\Phi_i(t),\\ &\Phi_i(t) = \Gamma_i(t) + \Theta_i(t), \quad i = 1,2,\end{aligned} \tag{S2}$$

where

$$\begin{aligned}\nu_i(a_i,\phi_i) &= \left[\left(\omega_{0i}^2 + 3\tilde{\beta}_i a_i^2/4\right)\left(1 + \lambda_i\cos 2\phi_i\right)\right]^{1/2},\\ \lambda_i &= \tilde{\beta}_i a_i^2 / 4\left(\omega_{0i}^2 + 3\tilde{\beta}_i a_i^2/4\right).\end{aligned} \tag{S3}$$

A_i, Γ_i, ν_i and Θ_i are stochastic processes. A_i and ν_i are the amplitude and instantaneous frequency of the *i*-th oscillator of the system (S1), respectively. ν_i can be approximated by the following Fourier series with a relative error

$$\nu_i(a_i,\phi_i) \approx b_{i0}(a_i) + b_{i2}(a_i)\cos 2\phi_i + b_{i4}(a_i)\cos 4\phi_i + b_{i6}(a_i)\cos 6\phi_i, \tag{S4}$$

where

$$
\begin{aligned}
b_{i0}(a_i) &= \left(\omega_{0i}^2 + 3\tilde{\beta}_i a_i^2/4\right)^{1/2}\left(1-\lambda_i^2/16\right),\\
b_{i2}(a_i) &= \left(\omega_{0i}^2 + 3\tilde{\beta}_i a_i^2/4\right)^{1/2}\left(\lambda_i/2+3\lambda_i^3/64\right),\\
b_{i4}(a_i) &= \left(\omega_{0i}^2 + 3\tilde{\beta}_i a_i^2/4\right)^{1/2}\left(-\lambda_i^2/16\right),\\
b_{i6}(a_i) &= \left(\omega_{0i}^2 + 3\tilde{\beta}_i a_i^2/4\right)^{1/2}\left(\lambda_i^3/64\right).
\end{aligned}
\tag{S5}
$$

Consequently, the averaged frequency $\omega_i(A_i)$ of the ith oscillator of the system (S1) can be approximated by $b_{i0}(a_i)$. Then the following approximation relationships exist in the subsequent analysis:

$$
\Phi_i(t) \approx \omega_i(A_i)t + \Theta_i(t) \tag{S6}
$$

Substitute Eq. (S2) into system (S1), one can obtain the following equations for A_i and Φ_i

$$
\begin{aligned}
\frac{dA_i}{dt} &= F_i^{(1)}(\mathbf{A},\boldsymbol{\Phi}) + G_{ik}^{(1)}(A_i,\Phi_i)\xi_k(t),\\
\frac{d\Phi_i}{dt} &= v_i(A_i,\Phi_i) + F_i^{(2)}(\mathbf{A},\boldsymbol{\Phi}) + G_{ik}^{(2)}(A_i,\Phi_i)\xi_k(t),
\end{aligned}
\tag{S7}
$$

where

$$
\begin{aligned}
F_i^{(1)} &= \frac{-A_i}{\omega_{0i}^2 A_i + \beta_i A_i^3} d_i(\boldsymbol{A},\boldsymbol{\Phi},t)\nu_i(A_i,\boldsymbol{\Phi}_i)\sin\boldsymbol{\Phi}_i,\\
F_i^{(2)} &= \frac{-1}{\omega_{0i}^2 A_i + \beta_i A_i^3} d_i(\boldsymbol{A},\boldsymbol{\Phi},t)\nu_i(A_i,\boldsymbol{\Phi}_i)\cos\boldsymbol{\Phi}_i,\\
G_{i1}^{(1)} &= \frac{-A_i}{\omega_{0i}^2 A_i + \beta_i A_i^3}\nu_i(A_i,\boldsymbol{\Phi}_i)\sin\boldsymbol{\Phi}_i, \qquad G_{i2}^{(1)} = \frac{-A_i^2}{\omega_{0i}^2 A_i + \tilde{\beta}_i A_i^3}\nu_i(A_i,\boldsymbol{\Phi}_i)\sin\boldsymbol{\Phi}_i\cos\boldsymbol{\Phi}_i,\\
G_{i1}^{(2)} &= \frac{-1}{\omega_{0i}^2 A_i + \beta_i A_i^3}\nu_i(A_i,\boldsymbol{\Phi}_i)\cos\boldsymbol{\Phi}_i, \qquad G_{i2}^{(2)} = \frac{-A_i}{\omega_{0i}^2 A_i + \tilde{\beta}_i A_i^3}\nu_i(A_i,\boldsymbol{\Phi}_i)\cos^2\boldsymbol{\Phi}_i,\\
d_1(\boldsymbol{A},\boldsymbol{\Phi},t) &= -\eta_1 A_1\nu_1(A_1,\boldsymbol{\Phi}_1)\sin\boldsymbol{\Phi}_1 + E_1\cos\boldsymbol{\Phi}_1 + G_1 A_2\cos\boldsymbol{\Phi}_2,\\
d_2(\boldsymbol{A},\boldsymbol{\Phi},t) &= -\eta_2 A_2\nu_2(A_2,\boldsymbol{\Phi}_2)\sin\boldsymbol{\Phi}_2 + E_2\cos\boldsymbol{\Phi}_2 + G_2 A_1\cos\boldsymbol{\Phi}_1.
\end{aligned}
\tag{S8}
$$

We assumethat the average natural frequency $\omega_i(A_i)\left(i=1,2\right)$ of the 2-DOF system (S1) has the following internal resonance relationship

$$
\omega_1(A_1) - \omega_2(A_2) = \varepsilon. \tag{S9}
$$

Herein, ε is a small detuning parameter. We introduce a new variable

$$\Delta = \Phi_1 - \Phi_2. \tag{S10}$$

Replace Φ_1 with Δ, Φ_2 and combine with Eq. (S7) and Eq. (S10) can obtain the new Itô SDEs for system (S1)

$$\begin{aligned}
\frac{\mathrm{d}A_i}{\mathrm{d}t} &= F_{i1}^{(1)}(\mathbf{A}, \Delta, \Phi_2) + G_{ik}^{(1)}(\mathbf{A}, \Delta, \Phi_2)\xi_{ik}(t), \\
\frac{\mathrm{d}\Delta}{\mathrm{d}t} &= v_1(A_2, \Phi_2) - v_2(A_2, \Phi_2) + F_1^{(2)}(\mathbf{A}, \Delta, \Phi_2) - F_2^{(2)}(\mathbf{A}, \Delta, \Phi_2) \\
&\quad + \left(G_{11}^{(2)}(\mathbf{A}, \Delta, \Phi_2) - G_{21}^{(2)}(\mathbf{A}, \Delta, \Phi_2)\right)\xi_1(t) + \left(G_{12}^{(2)}(\mathbf{A}, \Delta, \Phi_2) - G_{22}^{(2)}(\mathbf{A}, \Delta, \Phi_2)\right)\xi_2(t) \\
\frac{\mathrm{d}\Phi_2}{\mathrm{d}t} &= v_2(A_2, \Phi_2) + F_2^{(2)}(\mathbf{A}, \Delta, \Phi_2) + G_{2k}^{(2)}(\mathbf{A}, \Delta, \Phi_2)\xi_k(t),
\end{aligned} \tag{S11}$$

$A_1(t), A_2(t), \Delta(t)$ are slowly varying variables while Φ_2 are rapidly varying variables. According to the Stratonovich-Khasminskii limit theorem (*62, 63*), $\left[\mathbf{A}^T, \Delta^T\right]^T$ weakly converge to a 3-dimensional Markov diffusion process. The average Itô SDEs governing A_1, A_2, Δ can be obtained by performing stochastic averaging and time averaging on the right-hand side of the equation for A_1, A_2, Δ in Eq. (S11)

$$\begin{aligned}
\mathrm{d}A_1 &= m_1(A_1, A_2, \Delta)\mathrm{d}t + \sigma_{11}(A_1, A_2)\mathrm{d}B_1(t) + \sigma_{12}(A_1, A_2)\mathrm{d}B_2(t), \\
\mathrm{d}A_2 &= m_2(A_1, A_2, \Delta)\mathrm{d}t + \sigma_{21}(A_1, A_2)\mathrm{d}B_1(t) + \sigma_{22}(A_1, A_2)\mathrm{d}B_2(t), \\
\mathrm{d}\Delta &= m_3(A_1, A_2, \Delta)\mathrm{d}t + \sigma_{31}(A_1, A_2)\mathrm{d}B_1(t) + \sigma_{32}(A_1, A_2)\mathrm{d}B_2(t).
\end{aligned} \tag{S12}$$

where the drift and diffusion coefficients are

$$\begin{aligned}
m_1(A_1, A_2, \Delta) = &-\frac{\eta_1 A_1\left(5\beta_1 A_1^2 / 4 + 2\omega_{01}^2\right) + G_1 A_2\left(2b_{10} - b_{12}\right)\sin\Delta}{4\left(\beta_1 A_1^2 + \omega_{01}^2\right)} \\
&+ \frac{D_1}{\pi}\left(\frac{\beta_1 A_1^2\left(5\beta_1 A_1^2 + 11\omega_{01}^2\right)}{16\left(\beta_1 A_1^2 + \omega_{01}^2\right)^3} + \frac{3\beta_1 A_1^2 + 4\omega_{01}^2}{8A_1\left(\beta_1 A_1^2 + \omega_{01}^2\right)^2}\right) \\
&+ \frac{D_2}{\pi}\left(\frac{A_1\omega_{01}^2\left(\beta_1 A_1 + 2\omega_{01}^2\right)}{16\left(\beta_1 A_1^2 + \omega_{01}^2\right)^3} + \frac{7\beta_1 A_1^2 + 8\omega_{01}^2}{32\left(\beta_1 A_1^2 + \omega_{01}^2\right)^2}\right)
\end{aligned}$$

$$
\begin{aligned}
m_2(A_1,A_2,\Delta) = &-\frac{\eta_2 A_2\left(5\beta_2 A_2^2/4+2\omega_{02}^2\right)+G_2A_1\left(b_{22}-2b_{20}\right)\sin\Delta}{4\left(\beta_2A_2^2+\omega_{02}^2\right)} \\
&+\frac{D_1}{\pi}\left(\frac{\beta_2A_2^2\left(5\beta_2A_2^2+11\omega_{02}^2\right)}{16\left(\beta_2A_2^2+\omega_{02}^2\right)^3}+\frac{3\beta_2A_2^2+4\omega_{02}^2}{8A_2\left(\beta_2A_2^2+\omega_{02}^2\right)^2}\right) \\
&+\frac{D_2}{\pi}\left(\frac{A_2\omega_{02}^2\left(\beta_2A_2+2\omega_{02}^2\right)}{16\left(\beta_2A_2^2+\omega_{02}^2\right)^3}+\frac{7\beta_2A_2^2+8\omega_{02}^2}{32\left(\beta_2A_2^2+\omega_{02}^2\right)^2}\right)
\end{aligned}
$$

$$
\begin{aligned}
m_3(A_1,A_2,\Delta) = \varepsilon &+\frac{-E_1\left(b_{12}+2b_{10}\right)+G_1A_2\left(b_{12}+2b_{10}\right)\cos\Delta}{4A_1\left(\alpha_1A_1^2+\omega_{01}^2\right)} \\
&-\frac{-E_2\left(b_{22}+2b_{20}\right)+G_2A_1\left(b_{22}+2b_{20}\right)\cos\Delta}{4A_2\left(\alpha_2A_2^2+\omega_{02}^2\right)}
\end{aligned}
$$

$$
\begin{aligned}
b_{11} &= \sigma_{1k}\sigma_{1k} = \frac{D_1}{\pi}\frac{5\beta_1A_1^2+8\omega_{01}^2}{16\left(\beta_1A_1^2+\omega_{01}^2\right)^3}+\frac{D_2}{\pi}\frac{3\beta_1A_1^2+4\omega_{01}^2}{32\left(\beta_1A_1^2+\omega_{01}^2\right)^2}, \\
b_{22} &= \sigma_{2k}\sigma_{2k} = \frac{D_1}{\pi}\frac{5\beta_2A_2^2+8\omega_{02}^2}{16\left(\beta_2A_2^2+\omega_{02}^2\right)^3}+\frac{D_2}{\pi}\frac{3\beta_2A_2^2+4\omega_{02}^2}{32\left(\beta_2A_2^2+\omega_{02}^2\right)^2}, \\
b_{33} &= \sigma_{3k}\sigma_{3k} = \frac{D_1}{\pi}\left(\frac{7\beta_1A_1^2+8\omega_{01}^2}{16A_1^2\left(\beta_1A_1^2+\omega_{01}^2\right)^2}+\frac{7\beta_2A_2^2+8\omega_{02}^2}{16A_2^2\left(\beta_2A_2^2+\omega_{02}^2\right)^2}\right) \\
&\quad+\frac{D_2}{\pi}\left(\frac{11\beta_1A_1^2+12\omega_{01}^2}{32A_1^2\left(\beta_1A_1^2+\omega_{01}^2\right)^2}+\frac{7\beta_2A_2^2+8\omega_{02}^2}{32A_1^2\left(\beta_2A_2^2+\omega_{02}^2\right)^2}\right), \\
b_{ij} &= \sigma_{ik}\sigma_{jk} = 0\ (i\neq j),
\end{aligned}
\tag{S13}
$$

Averaged Itô SDEs (Eq. (S12)) is reduced to ordinary differential equation when the intensity of excitations $D_i = 0$. The stationary amplitude response curves of the system can be obtained by letting $\mathrm{d}A_1/\mathrm{d}t = \mathrm{d}A_2/\mathrm{d}t = \mathrm{d}\Delta/\mathrm{d}t = 0$.

S2. Simulation details

To investigate the synchronization behavior and noise-induced dynamics of two coupled nonlinear oscillators, we numerically integrate the reduced stochastic system derived in Eq. (S12), which governs the slow evolution of the modal amplitudes $A_1(t)$, $A_2(t)$ and phase difference $\Delta(t)$. These equations result from applying the stochastic averaging method to the original high-dimensional system, and capture the effective Markovian dynamics on slow timescales (see derivation in Section S1). The dynamics of the original system can be effectively represented by the system of three coupled Itô stochastic differential equations (Eq. (S12)), and the explicit forms of the drift terms (m_1, m_2, and m_3) and diffusion terms (b_{11}, b_{22}, and b_{33}) are provided in Eq. (S13).

Numerical Implementation

We implemented this system using the Euler–Maruyama scheme, a standard numerical integrator for Itô SDEs. Through time discretization (time step Δt), Euler discretization expressions of Eq. (S12) is

$$\begin{aligned}
A_1(n\Delta t+\Delta t) &\approx A_1(n\Delta t)+m_1(A_1,A_2,\Delta)\big|_{t=n\Delta t}\Delta t+\sigma_{11}(A_1,A_2)\big|_{t=n\Delta t}\mathrm{d}B_1(t)+\sigma_{12}(A_1,A_2)\big|_{t=n\Delta t}\mathrm{d}B_2(t)\\
A_2(n\Delta t+\Delta t) &\approx A_2(n\Delta t)+m_2(A_1,A_2,\Delta)\big|_{t=n\Delta t}\Delta t+\sigma_{21}(A_1,A_2)\big|_{t=n\Delta t}\mathrm{d}B_1(t)+\sigma_{22}(A_1,A_2)\big|_{t=n\Delta t}\mathrm{d}B_2(t)\\
\Delta(n\Delta t+\Delta t) &\approx \Delta(n\Delta t)+m_3(A_1,A_2,\Delta t)\big|_{t=n\Delta t}\mathrm{d}t+\sigma_{31}(A_1,A_2)\big|_{t=n\Delta t}\mathrm{d}B_1(t)+\sigma_{32}(A_1,A_2)\big|_{t=n\Delta t}\mathrm{d}B_2(t).
\end{aligned} \tag{S14}$$

where $dB(t)$ is standard Brownian motion, and its value is obtained from the following equation

$$dB(t)=B(n\Delta t)-B((n-1)\Delta t)=\left(\frac{\xi_n}{\sqrt{\Delta t}}\right)\Delta t \tag{S15}$$

where ξ_n is a Gaussian random number with zero mean and unit variance.

Parameters are set as follows:

- Natural frequency: $\omega_{01}=\omega_{02}=1$,
- Linear damping: $\eta_1=\eta_2=0.3$,
- Quadratic nonlinearity: $\alpha_1=\alpha_2=0.01$,
- Cubic nonlinearity: $\beta_1=\beta_2=0.5$,
- Feedback strength: $E_1=E_2=0.2$,
- Coupling strength: $G_1=G_2=0.01$,
- Stochastic noise intensities: $D_1=D_2=D$.

Numerical method

Simulations were performed over the interval $t \in [0,\ 400]$ with a fixed time step $\Delta t = 0.1$, yielding $N = 4001$ time steps. The initial conditions were: $A_1(0) = 1.2$, $A_2(0) = 1.0$, $\Delta(0) = \frac{\pi}{2}$. The drift terms m_1, m_2, m_3 are nonlinear functions of A_1, A_2, Δ and are constructed by evaluating the expressions derived in Eq. (S13), which include nonlinear self- and cross-coupling terms, and various noise-induced modulation components. The coefficients σ_{ij} are computed based on the second-order noise moments and depend on both the noise intensities D_1, D_2 and system parameters (e.g., damping, stiffness nonlinearity, and frequency detuning).

We performed simulations for different values of the noise strength D_1 and D_2. For each value, the stochastic trajectories of $A_1(t)$, $A_2(t)$, $\Delta(t)$ were computed, and system frequencies were calculated from the first equation in Eq. (S5), i.e.,

$$\omega_1(t) = b_{10}\left(A_1(t)\right),\ \omega_2(t) = b_{20}\left(A_2(t)\right). \tag{S16}$$

which are used as references for identifying synchronized states. Deviations from this equilibrium under noise quantify the robustness of synchronization and noise-induced desynchronization or modulation.

MATLAB Code Structure

The simulation was implemented in MATLAB. At each time step, the following steps were performed:

1. Discretize the averaged Itô SDEs (S12),
2. Compute drift and diffusion terms based on Eq. (S13),
3. Generate stochastic increments $\mathrm{d}B_1, \mathrm{d}B_2$,
4. Apply Euler-Maruyama update to A_1, A_2, Δ,
5. Calculate the system frequencies by Eq. (S16).

S3. General relationship between the phase delay and oscillation frequency

The normalized governing equation for the nonlinear oscillator with phase delay feedback force is (*9, 28*)

$$\ddot{x}+Q^{-1}\dot{x}+x+\beta x^3=f_0\cos\left(\phi+\phi_0\right) \tag{S17}$$

The analytical solution of Eq. (S14) can be obtained using the harmonic approximation,

$$x=A\cos\left(\phi\right) \tag{S18}$$

$$\dot{x}=-A\Omega\sin\left(\phi\right) \tag{S19}$$

Substituting Eqs. (S15) and (S16) into Eq. (S14), we have

$$-A\Omega^2\cos\left(\phi\right)-Q^{-1}A\Omega\sin\left(\phi\right)+A\cos\left(\phi\right)+\beta A^3\cos^3\left(\phi\right)=f_0\cos\left(\phi+\phi_0\right) \tag{S20}$$

Neglecting higher-order harmonic contributions, we have the approximate expression (*64*)

$$\cos^3\left(\phi\right)\approx\frac{3}{4}\cos\left(\phi\right) \tag{S21}$$

Substituting Eq. (S18) into Eq. (S17) and separating terms in proportion to $\cos\left(\phi\right)$ and $\sin\left(\phi\right)$, respectively, we get two equations which can be merged into a single equation in the complex domain

$$\left[\left(1-\Omega^2\right)A+\frac{3}{4}\beta A^3-f_0\cos\left(\phi_0\right)\right]+\left[A\Omega Q^{-1}+f_0\sin\left(\phi_0\right)\right]\mathrm{i}=f_s e^{-\mathrm{i}\phi_s} \tag{S22}$$

Making the following equations equal to 0, we have the self-sustained stable amplitude and frequency as

$$A_0=\frac{Qf_0\sin\left(\phi_0\right)}{\Omega_0}$$

$$\Omega_0=\frac{1}{\sqrt{2}}\left(1+\left(1+3\beta Q^2 f_0^2\sin^2\left(\phi_0\right)\right)^{\frac{1}{2}}\right)^{\frac{1}{2}} \tag{S23}$$

S4. <u>Frequency tuning via electrothermal adjustment</u>

The two resonators exhibit a non-negligible mismatch in their natural frequencies due to the small differences in device dimensions, which hinders synchronization. To address this mismatch, the electrothermal effect was employed to tune the natural frequencies. By injecting electrothermal current into the beams, the resulting heating induces an axial force into the beam and thus causes a frequency shift. As shown in Fig. S4A, the linear amplitude-frequency responses of R1 and R2, measured at various I_{th}, illustrate how the natural frequencies shift with the injected electrothermal current, where the natural frequencies are extracted from the Lorentz fitting, as shown in Fig. S4B.

Fig. S4C presents the extracted relationship between the natural frequencies of both resonators and the electrothermal current, indicating that the natural frequencies decrease as the current increases. The solid lines represent quadratic fits, suggesting that the electrothermal effect on the natural frequencies follows the behavior described in (*65*). As the electrothermal current increased from 0 mA to 6 mA, the resistance remained nearly constant, indicating stable material properties within the operating range. The identical frequency range for both resonators is marked by the shaded pink area, indicating the operational range where the two resonators can achieve synchronization.

To confirm that heating does not alter the fundamental properties of the resonators, we measured the resistance of two resonators under varying electrothermal currents, as shown in Fig. S4D. The experimental data (dots) and their linear fits (solid lines) demonstrate that the electrothermal current has no detrimental impact on the resonators' fundamental properties.

S5. Parameters identification

The two beams are almost identical and should have approximately the same parameters. The generalized equation of motion, which considers the first three orders of nonlinear damping coefficients and stiffness nonlinearities, is (*66, 67*):

$$M\ddot{X} + C_1\dot{X} + C_2\dot{X}^2 + C_3\dot{X}^2 + N_1X^2\dot{X} + K_1X + K_2X^2 + K_3X^3 = F\,.$$

This equation can approximately identify the system parameters through the amplitude frequency curves (**Fig. 2B**) fitting. The identified parameters are listed in Table S1.

S6. Establishing self-oscillation

The self-oscillation is achieved based on the built-in phase-locked loop (PLL), where the schematic diagram is depicted in Fig. S5A. We demonstrated self-oscillation in two different regimes: linear and nonlinear response. The linear open-loop *a-f* and phase-frequency (*p-f*) responses are shown in Fig. S5B, with an excitation magnitude of V_{AC} = 50 mV. A phase-locked loop was used to implement feedback control, locking the frequency and phase at approximately 270.08 kHz and $\pi/2$ (marked by yellow dots), respectively. For a linear resonator, phase locking near $\pi/2$ ensures maximum signal-to-noise ratio (SNR) (*27*). The time-domain amplitude response obtained from the closed-loop system is shown in Fig. S5C, displaying typical periodic oscillations.

The periodic self-oscillation can be fully characterized by its evolution in phase space, represented by the coordinates of oscillation amplitude and velocity $(x, \dot{x})$, where x is the vibration amplitude. As shown in Fig. S5D, the limit cycle forms a nearly closed loop, indicating a well-established self-oscillation state. However, due to the low oscillation amplitude and resulting low SNR, the response deviates slightly from a perfect circle, with some fluctuations induced by noise.

We further investigated nonlinear self-oscillations by increasing the excitation strength (V_{AC} = 500 mV) to drive the resonator into the nonlinear regime. The nonlinear *a-f* and *p-f* responses are shown in Fig. S5E, where forward (blue lines) and backward (red lines) sweeps reveal hysteresis due to the nonlinearity. The response was locked at a relatively high amplitude to achieve a larger SNR. The resulting time-domain oscillation amplitude (Fig. S5F) exhibits a more complex pattern compared to Fig. S5C, due to the asymmetric characteristics associated with odd-order nonlinearities, such as cubic (Duffing) nonlinearity. In the phase space representation, the limit cycle forms two distinct closed curves, as shown in Fig. S5G, contrasting with the single closed loop observed in the linear case (Fig. S5D). Despite this difference, both limit cycles confirm the presence of well-established self-oscillations in both the linear and nonlinear regimes.

Supplementary figures

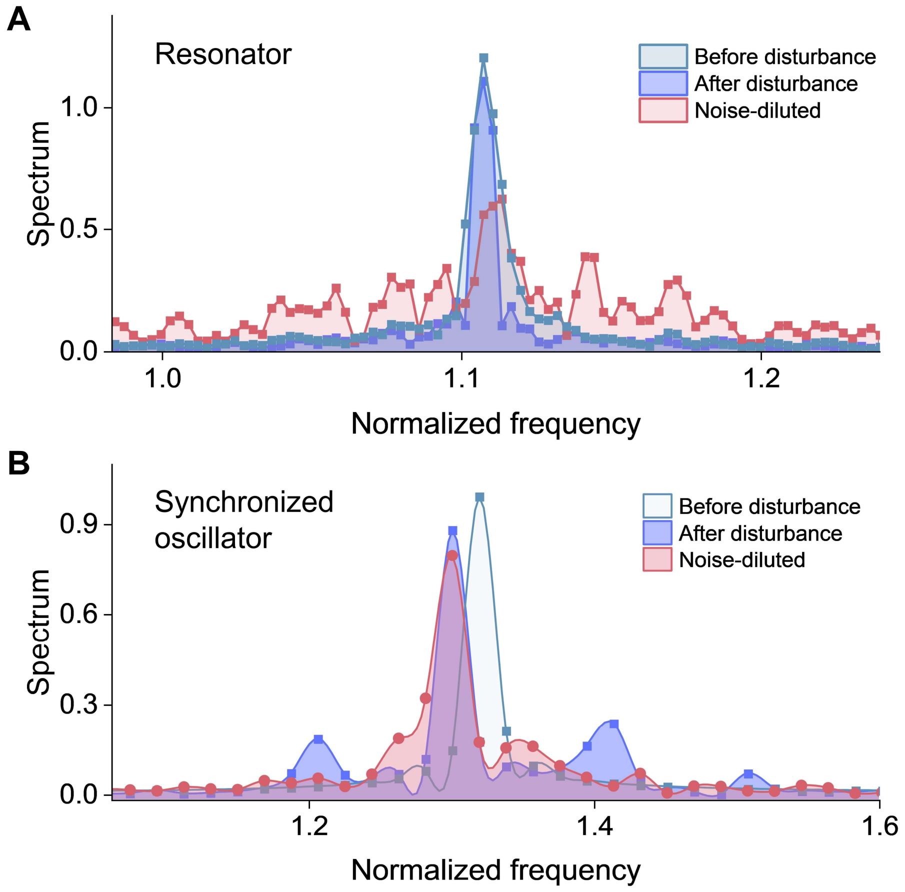

Fig. S1. Simulation of the noise effects on the resonator and synchronized oscillator before and after disturbance. (**A**) and (**B**) are the simulations of the uncoupled resonator and the synchronized oscillator before and after the disturbances.

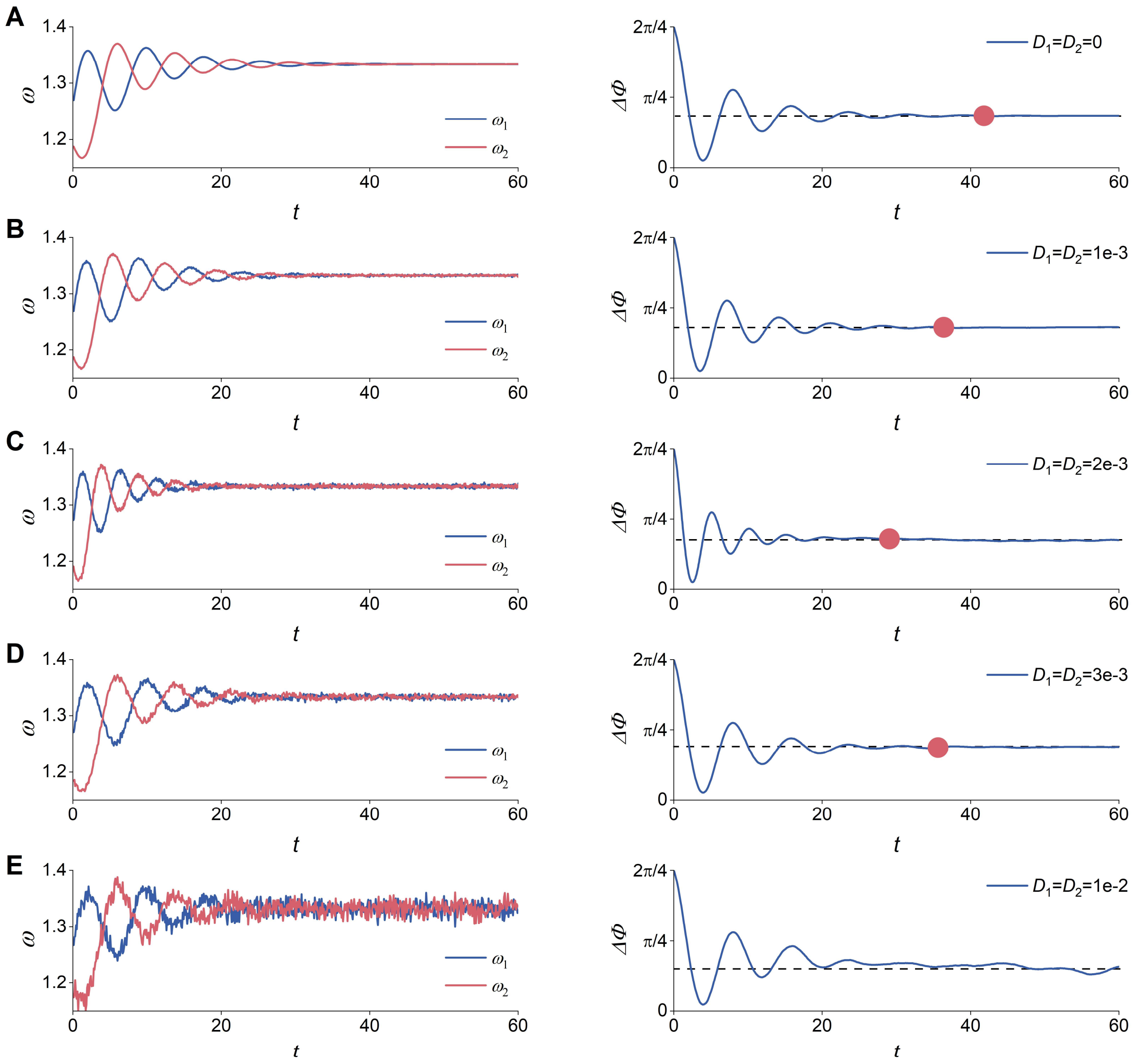

Fig. S2. Simulated time evolution of two coupled oscillators' non-dimensional frequencies (*ω*) and phase difference (*Δ*) under varying noise intensities (*D*). Time series plots show the instantaneous frequencies ($\omega_1(A)$, $\omega_2(A)$), amplitudes (A_1, A_2), and phase difference (Δ) for different noise intensities. Numerical integration was performed using MATLAB over a simulation duration of 400 seconds (here we show 60 seconds). Initial conditions were set as $A_{10} = 1.2$, $A_{20} = 1.0$, $\Delta_0 = \pi / 2$, with system parameters $\eta_1 = \eta_2 = 0.3$, $\alpha_1 = \alpha_2 = 0.01$, $\beta_1 = \beta_2 = 0.5$, $\omega_{01} = \omega_{02} = 1$, $E_1 = E_2 = 0.2$. **(A)**–**(E)** correspond to different noise intensities applied equally to both oscillators ($D_1 = D_2 = D$, $D = 0$, $D = 1\times10^{-3}$, $D = 2\times10^{-3}$, $D = 3\times10^{-3}$ and $D = 1\times10^{-2}$, respectively).

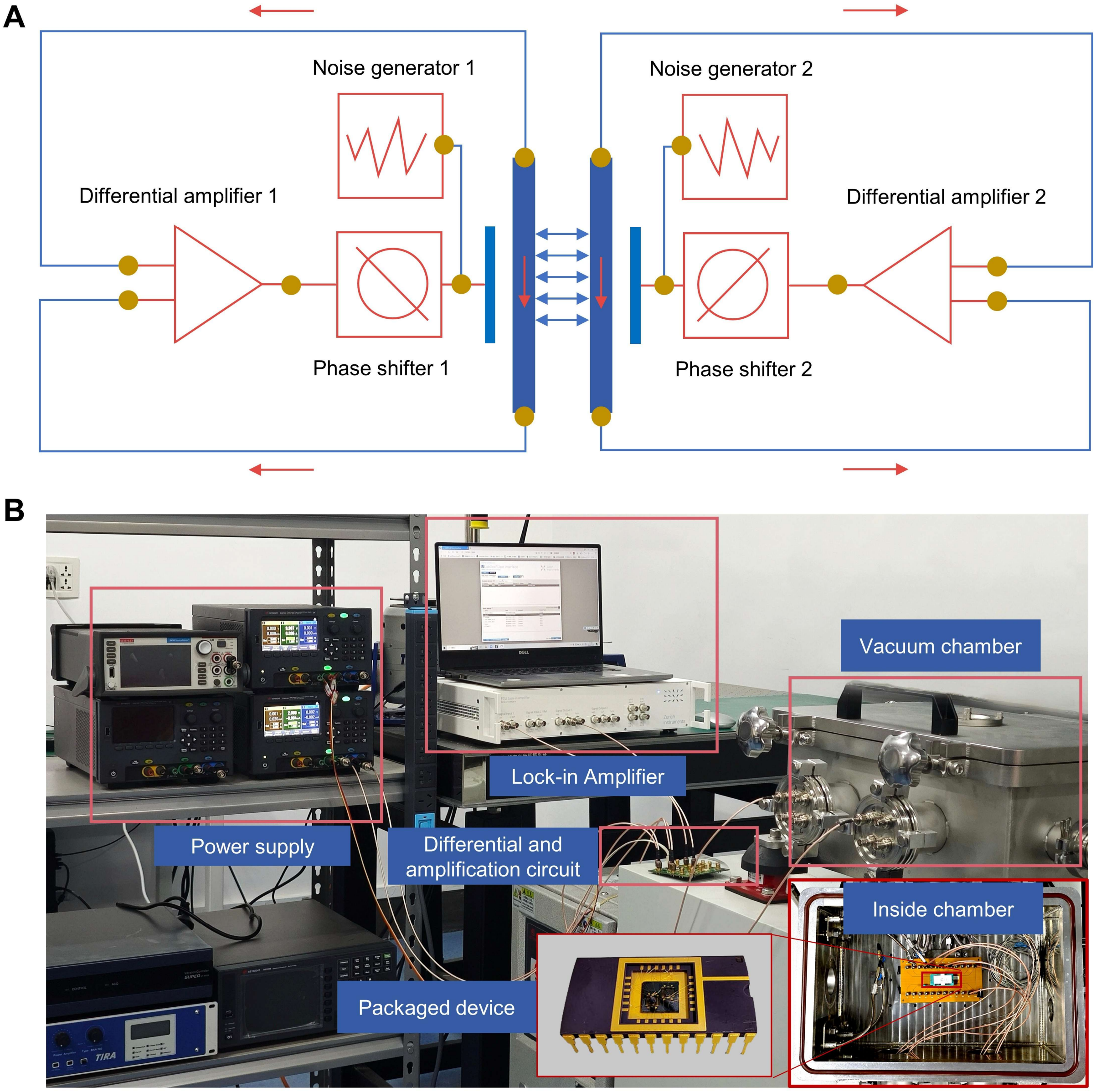

Fig. S3. Experimental setup of the microscale characterizations. (**A**) Experimental setup of the excitation and detection circuit of the micromechanical oscillators. (**B**) Experimental stand, where the two insets show the packaged device and inside view of the vacuum chamber, respectively.

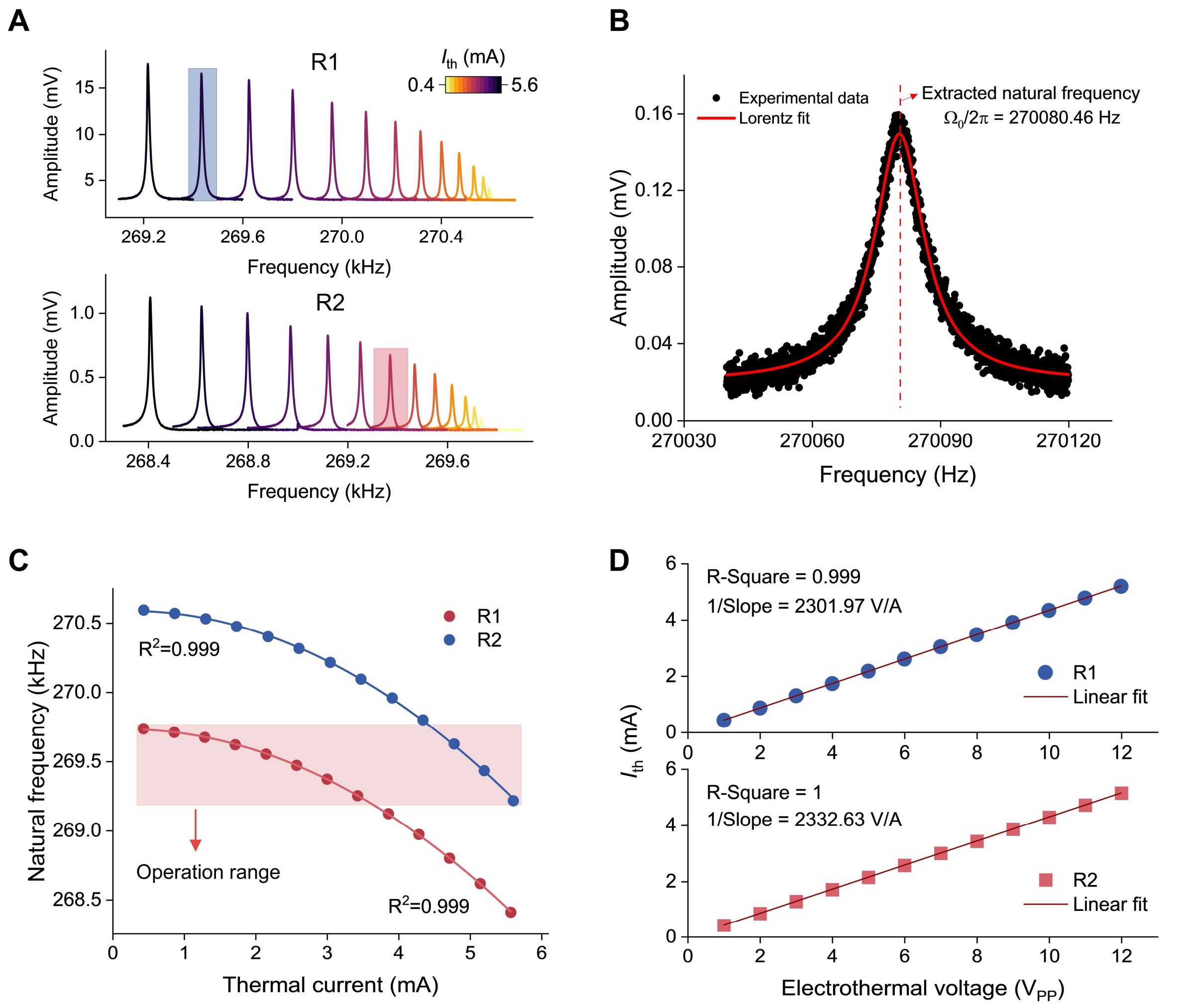

Fig. S4. Frequency tuning via electrothermal adjustment. (**A**) Amplitude-frequency responses of the two oscillators at various electrothermal currents, ranging from 0.4 to 5.6 mA, under an AC excitation of $V_{AC} = 50$ mV. (**B**) Resonance properties characterized using Lorentzian fitting, where black dots represent experimental data, the red solid line is the Lorentzian fit, and the red dashed line indicates the calculated natural frequency. (**C**) Operational domain of the coupled oscillators, with the pink region denoting identical natural frequencies for both oscillators, as determined by Lorentzian fitting. (**D**) Current responses of the two resonators at different electrothermal currents, with dots representing experimental results and solid lines showing the linear fit.

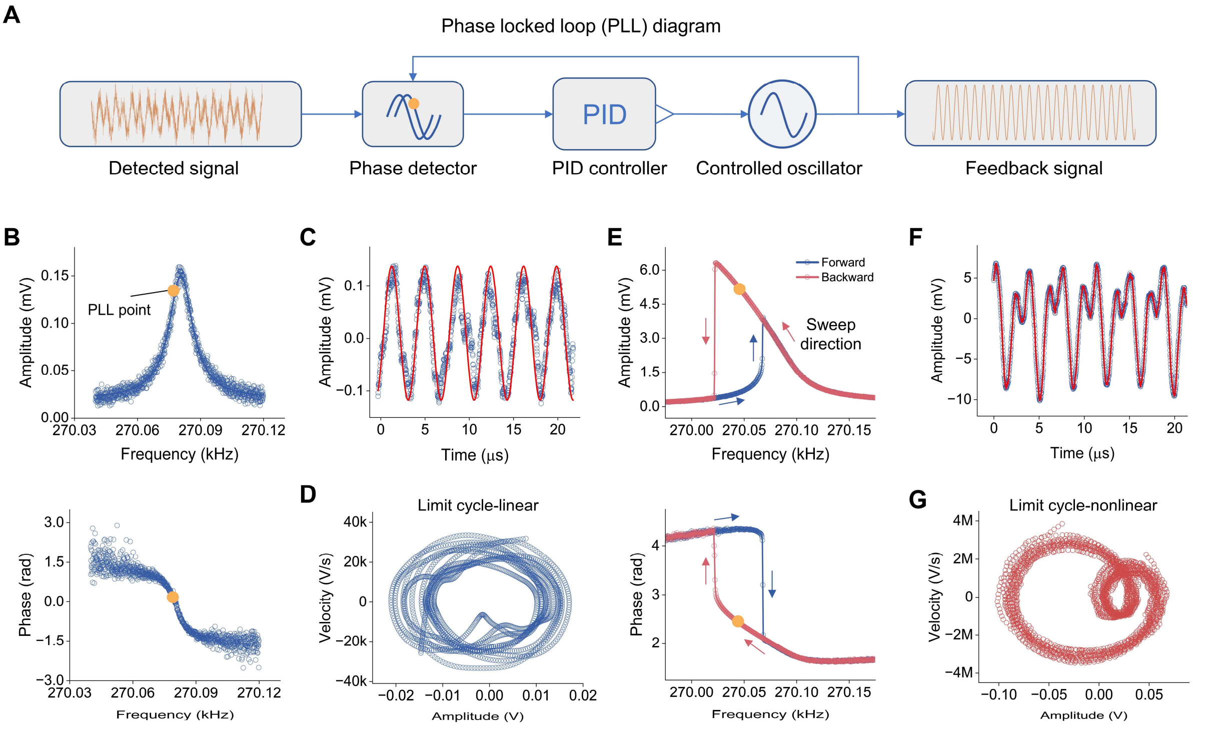

Fig. S5. Device characteristics and experimental setup. (**A**) Schematic diagram of the phase-locked loop (PLL). (**B**) Amplitude-frequency response and phase-frequency responses of resonator 1 at linear state, where the yellow dot is the locking point during the stable phase-locking condition maintained by the PLL feedback control. (**C**) Measured corresponding time domain response of the resonator at the locking point. (**D**) Limit cycle of the response working in the linear state. (**E**) Amplitude-frequency and phase-frequency responses of the resonator 1 working in a nonlinear state. (**F**) and (**G**) are the time-domain amplitude and limit cycle corresponding to the resonator locking in the yellow points.

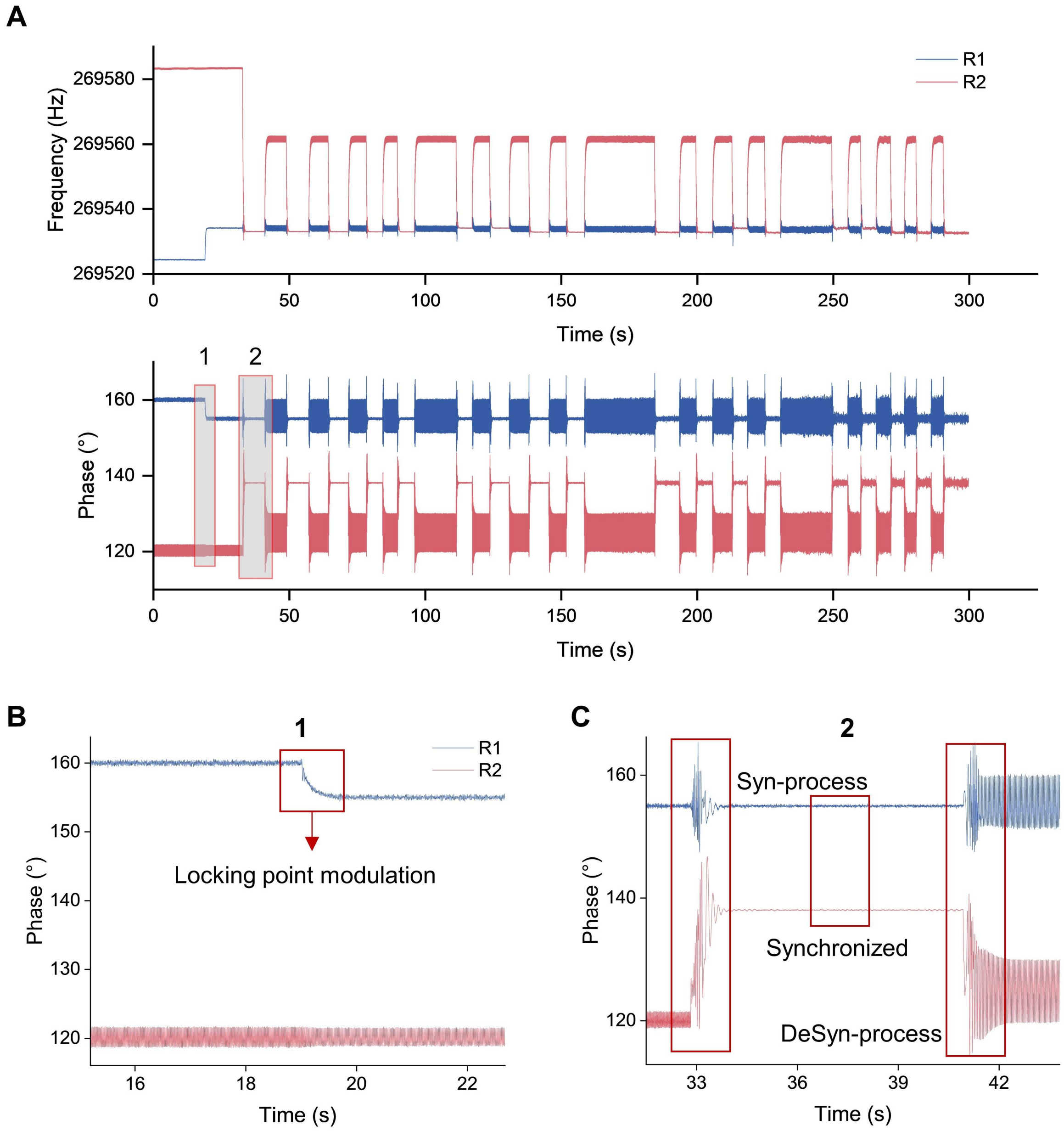

Fig. S6. Phase dynamics under various nonlinear conditions. (**A**) Time evolution of the frequency and phase responses of R1 (red) and R2 (blue) under multiple conditions, including phase locking point modulation, frequency switching, synchronization, and desynchronization. Modulation of the locking point triggers distinct transitions among these dynamic states, as shown across multiple trials. (**B**) Zoomed-in view of region **1** in (A), showing detailed phase evolution during locking point modulation-induced phase transition. (**C**) Zoomed-in view of region **2** in (A), highlighting the full phase trajectory across synchronization onset (Syn-process), synchronized state, and desynchronization onset (DeSyn-process).

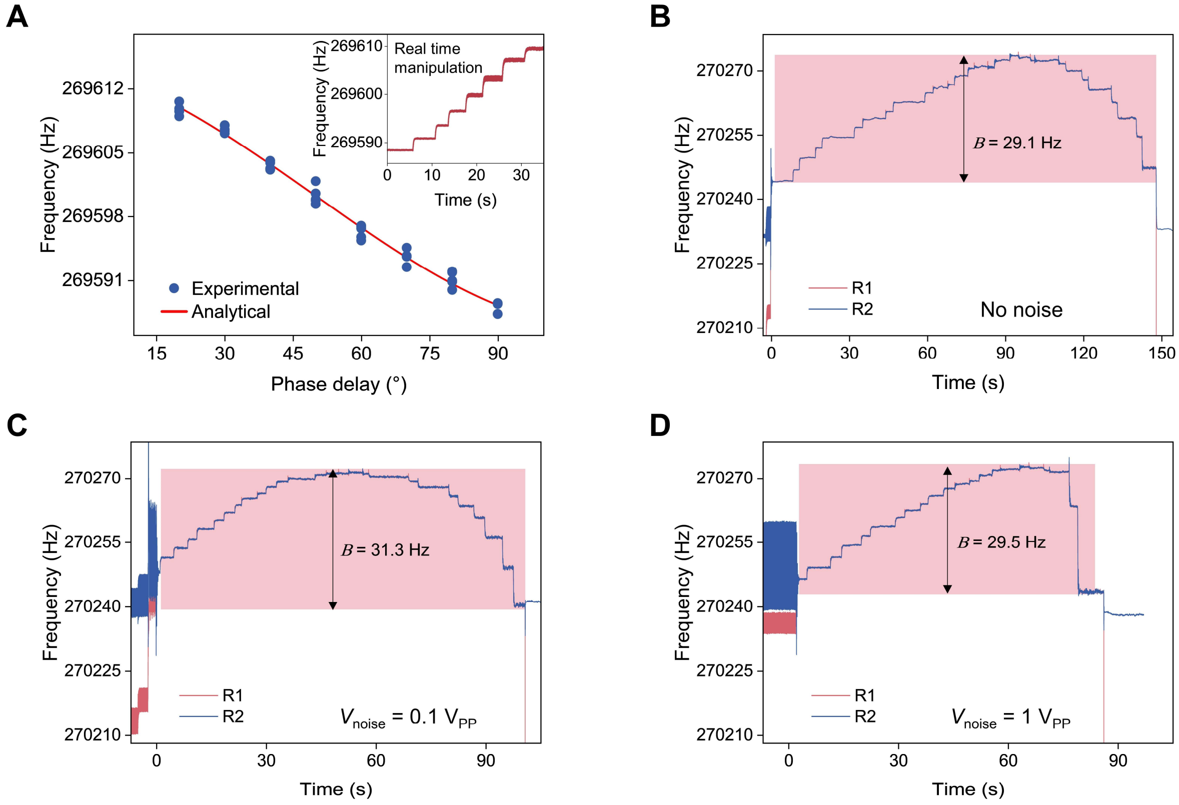

Fig. S7. Feedback control and synchronization region characterization. (**A**) Frequency modulation achieved through phase delay tuning, with blue dots representing experimental measurements and the red line indicating analytical results. The inset shows the real-time frequency response under phase delay modulation. (**B**) Measured synchronization region without noise, where the shaded pink area highlights the synchronization region, measured to be 29.1 Hz. (**C**) and (**D**) show the measured synchronization regions with noise intensities of 0.1 V_{PP} and 1 V_{PP}, respectively. Both noises operate up to a frequency of 10 MHz, with measured synchronization bandwidths of 31.3 Hz and 29.5 Hz, respectively.

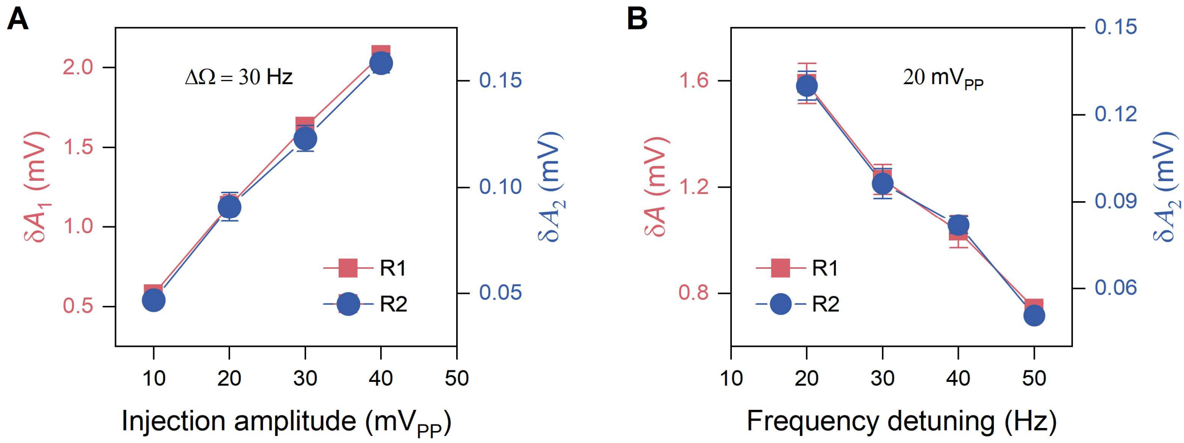

Fig. S8. Effects of external disturbances on oscillator amplitude fluctuations. (**A**) Amplitude fluctuations of both oscillators as a function of the injected signal amplitude at a fixed frequency detuning of $\Delta\Omega = 30$ Hz. (**B**) Amplitude fluctuations as a function of frequency detuning between the injected signal and the oscillators, with a fixed injection amplitude of 20 mV_{PP}. In both cases, larger injection amplitudes and smaller frequency detuning result in stronger amplitude fluctuations.

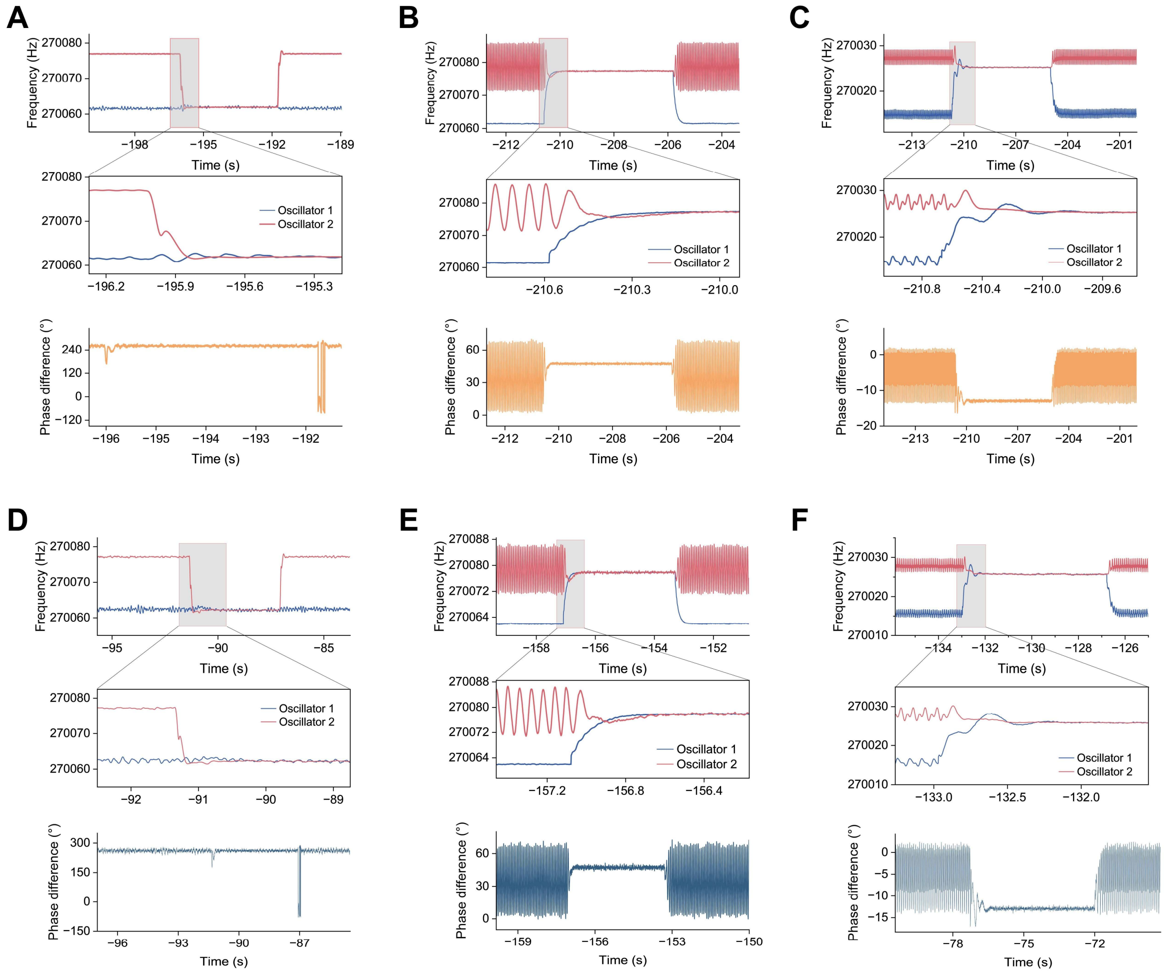

Fig. S9. Synchronization processes without and with the noise effects. (A) Synchronization processes of the linear oscillator with linear oscillator without noise, where the upper, middle and lower panels are the time domain frequency, zoomed-in synchronization process, and the phase difference in time scale. **(B)** Synchronization processes of the linear oscillator with a nonlinear oscillator without noise, where the upper, middle and lower panels are the time domain frequency, zoomed-in synchronization process and the phase difference in time scale. **(C)** Synchronization processes of the nonlinear oscillator with a nonlinear oscillator without noise, where the upper, middle and lower panels are the time domain frequency, zoomed-in synchronization process and the phase difference in time scale. **(D), (E)** and **(F)** correspond with the modulation of the noise.

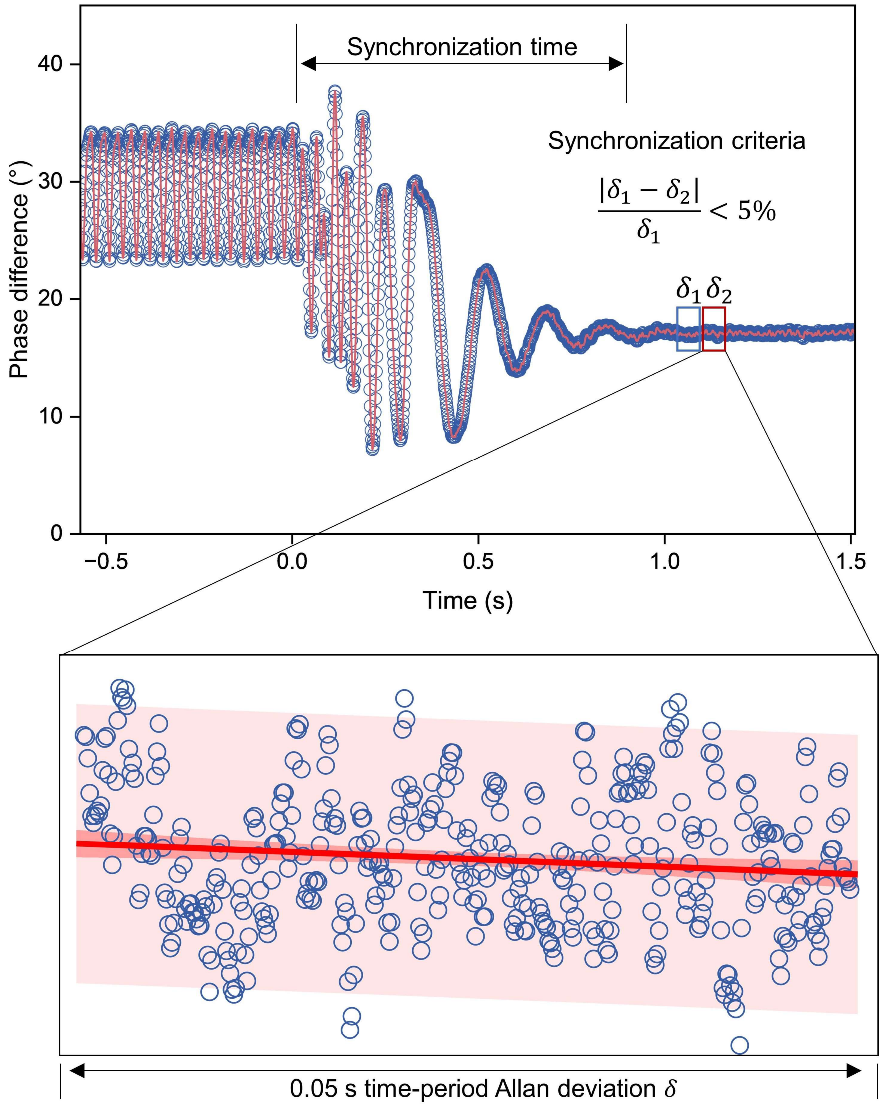

Fig. S10. Characterization of the synchronization time. This figure illustrates the phase difference between two oscillators during the synchronization process. Synchronization is identified when the measured Allan deviation error at a period 0.1 s, compared to the preceding 0.1 s, is less than 5%.

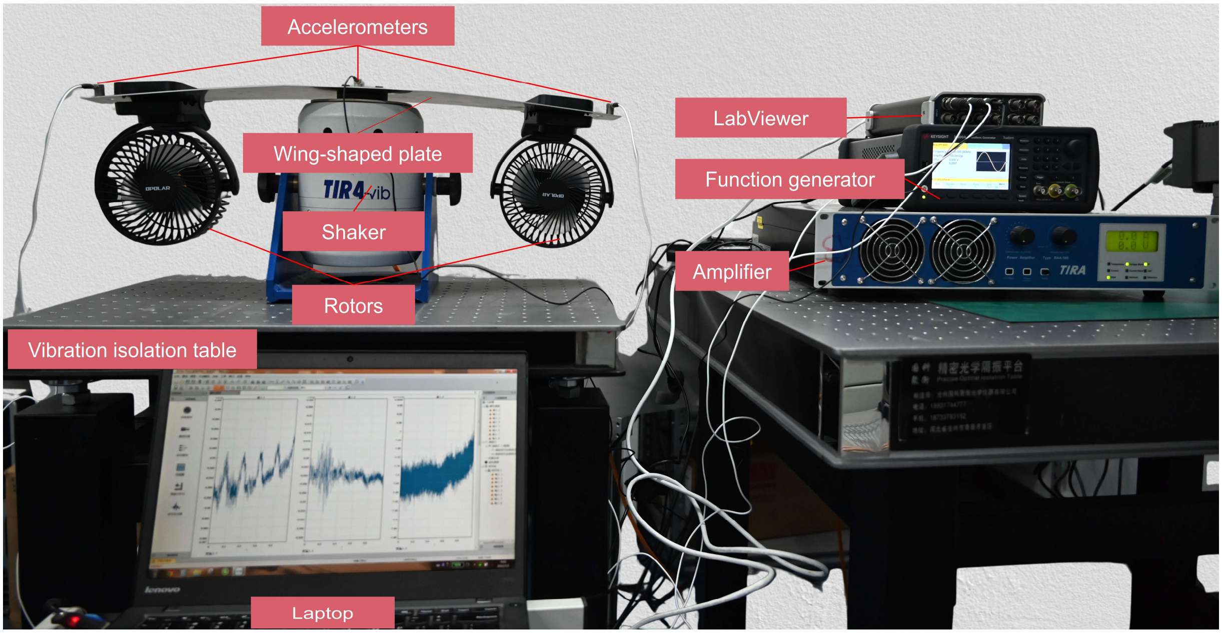

Fig. S11. Experimental setup for characterization of the macro rotors. The rotors are mounted on a wing-shaped aluminum alloy plate that is attached to a vibrator placed on a vibration isolation table. An accelerometer measures the wing's vibrations, with the recorded signal processed using LabVIEW. Noise Both noise and impact signals are generated by a function generator, then amplified and sent to the electrodynamic actuator (shaker).

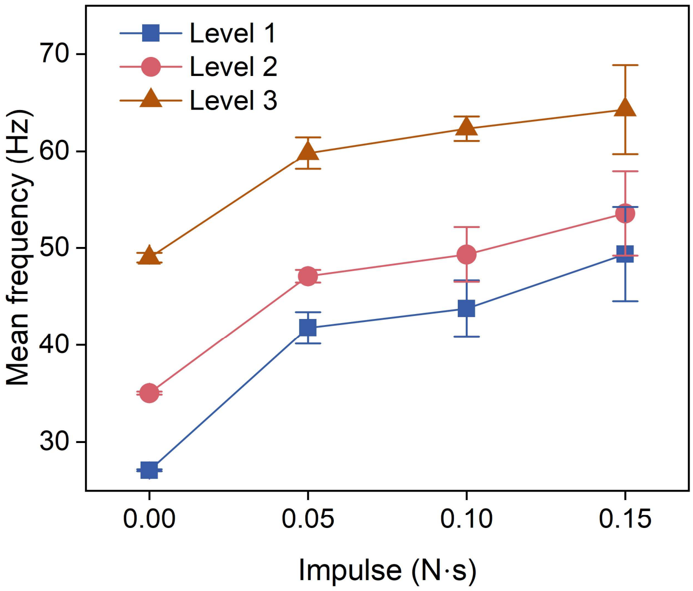

Fig. S12. Impulse-induced modulation of rotor oscillation frequency. Mean oscillation frequency as a function of applied impulse for three rotor levels (Level 1, Level 2 and Level 3). All levels exhibit a monotonic increase in frequency with impulse. Error bars denote standard deviation over repeated measurements.

Supplementary Table

Table S1. Characterized parameters of the electrostatically coupled micro-resonators

**Parameters**	**Values**
Equivalent mass M	4.2×10^{-7}
1st order damping coefficient C_1	1.8×10^{-5}
2nd order damping coefficient C_2	2.1×10^{-16}
3rd order damping coefficient C_3	2.3×10^{-20}
Nonlinear damping coefficient N_1	2.18×10^{-22}
1st order stiffness K_1	1.48×10^{6}
2nd order stiffness K_2	-3.8×10^{-4}
3rd order stiffness K_3	-3.1×10^{1}

REFERENCES AND NOTES

1. L. Glass, Synchronization and rhythmic processes in physiology. *Nature* **410**, 277–284 (2001).

2. S. Shahal, A. Wurzberg, I. Sibony, H. Duadi, E. Shniderman, D. Weymouth, N. Davidson, M. Fridman, Synchronization of complex human networks. *Nat. Commun.* **11**, 3854 (2020).

3. I. Nitsan, S. Drori, Y. E. Lewis, S. Cohen, S. Tzlil, Mechanical communication in cardiac cell synchronized beating. *Nat. Phys.* **12**, 472–477 (2016).

4. R. E. Goldstein, M. Polin, I. Tuval, Noise and synchronization in pairs of beating eukaryotic flagella. *Phys. Rev. Lett.* **103**, 168103 (2009).

5. C. Chen, S. Liu, X.-Q. Shi, H. Chaté, Y. Wu, Weak synchronization and large-scale collective oscillation in dense bacterial suspensions. *Nature* **542**, 210–214 (2017).

6. A. Japaridze, V. Struijk, K. Swamy, I. Rosłoń, O. Shoshani, C. Dekker, F. Alijani, Synchronization of *E. coli* bacteria moving in coupled microwells. *Small* **21**, e2407832 (2025).

7. Y. Ito, T. I. Shiramatsu, N. Ishida, K. Oshima, K. Magami, H. Takahashi, Spontaneous beat synchronization in rats: Neural dynamics and motor entrainment. *Sci. Adv.* **8**, eabo7019 (2022).

8. V. M. Vinokur, T. I. Baturina, M. V. Fistul, A. Yu. Mironov, M. R. Baklanov, C. Strunk, Superinsulator and quantum synchronization. *Nature* **452**, 613–615 (2008).

9. D. Antonio, D. A. Czaplewski, J. R. Guest, D. López, S. I. Arroyo, D. H. Zanette, Nonlinearity-induced synchronization enhancement in micromechanical oscillators. *Phys. Rev. Lett.* **114**, 034103 (2015).

10. J. L. Wilson, A. A. Pahlavan, M. A. Erinin, C. Duprat, L. Deike, H. A. Stone, Aerodynamic interactions of drops on parallel fibres. *Nat. Phys.* **19**, 1667–1672 (2023).

11. E. Um, M. Kim, H. Kim, J. H. Kang, H. A. Stone, J. Jeong, Phase synchronization of fluid-fluid interfaces as hydrodynamically coupled oscillators. *Nat. Commun.* **11**, 5221 (2020).

12. M. H. Matheny, J. Emenheiser, W. Fon, A. Chapman, A. Salova, M. Rohden, J. Li, M. H. de Badyn, M. Pósfai, L. Duenas-Osorio, M. Mesbahi, J. P. Crutchfield, M. C. Cross, R. M. D'Souza, M. L. Roukes, Exotic states in a simple network of nanoelectromechanical oscillators. *Science* **363**, eaav7932 (2019).

13. D. Shenhav Feigin, O. Shoshani, Synchronization of non-weakly coupled aeroelastic oscillators. *Commun. Phys.* **7**, 211 (2024).

14. S. B. Shim, M. Imboden, P. Mohanty, Synchronized oscillation in coupled nanomechanical oscillators. *Science* **316**, 95–99 (2007).

15. M. H. Matheny, M. Grau, L. G. Villanueva, R. B. Karabalin, M. C. Cross, M. L. Roukes, Phase synchronization of two anharmonic nanomechanical oscillators. *Phys. Rev. Lett.* **112**, 014101 (2014).

16. C. Spiess, S. Töpfer, S. Sharma, A. Kržič, M. Cabrejo-Ponce, U. Chandrashekara, N. L. Döll, D. Rieländer, F. Steinlechner, Clock synchronization with correlated photons. *Phys. Rev. Appl.* **19**, 054082 (2023).

17. X. Cui, V. Mylnikov, P. Johansson, M. Käll, Synchronization of optically self-assembled nanorotors. *Sci. Adv.* **10**, eadn3485 (2024).

18. A. Greilich, N. E. Kopteva, V. L. Korenev, P. A. Haude, M. Bayer, Exploring nonlinear dynamics in periodically driven time crystal from synchronization to chaotic motion. *Nat. Commun.* **16**, 2936 (2025).

19. H. Y. Luan, Y. H. Ouyang, Z. W. Zhao, W. Z. Mao, R. M. Ma, Reconfigurable moiré nanolaser arrays with phase synchronization. *Nature* **624**, 282–288 (2023).

20. X. Xiong, C. Wu, F. Blaabjerg, An improved synchronization stability method of virtual synchronous generators based on frequency feedforward on reactive power control loop. *IEEE Trans. Power Electron.* **36**, 9136–9148 (2021).

21. M. Zou, P. Fang, Y. Hou, H. Peng, Investigation on multiple-frequency synchronization experiment of vibration system with dual-rotor actuation. *Mech. Syst. Signal Process.* **164**, 108261 (2022).

22. M. Sueda, H. Mori, T. Kondou, Analytical study of self-synchronization in two unbalanced rotors based on energetic conditions. *J. Sound Vib.* **521**, 116618 (2022).

23. L. Beltramelli, A. Mahmood, P. Österberg, M. Gidlund, P. Ferrari, E. Sisinni, Energy efficiency of slotted LoRaWAN communication with out-of-band synchronization. *IEEE Trans. Instrum. Meas.* **70**, 5501211 (2021).

24. S. Watt, M. Kostylev, A. B. Ustinov, Enhancing computational performance of a spin-wave reservoir computer with input synchronization. *J. Appl. Phys.* **129**, 044501 (2021).

25. O. Shoshani, D. Heywood, Y. Yang, T. W. Kenny, S. W. Shaw, Phase noise reduction in an MEMS oscillator using a nonlinearly enhanced synchronization domain. *J. Microelectromech. Syst.* **25**, 870–876 (2016).

26. B. Hu, L. Zhan, S. Sahoo, L. Chen, H. Nian, F. Blaabjerg, Synchronization stability analysis under ultra-weak grid considering reactive current dynamics. *IEEE Trans. Ind. Electron.* **71**, 15220–15223 (2024).

27. D. Pu, R. Huan, X. Wei, Frequency stability improvement for piezoresistive micromechanical oscillators via synchronization. *AIP Adv.* **7**, 035119 (2017).

28. Z. Shi, D. Pu, X. Wang, R. Huan, Z. Jiang, X. Wei, Phase-delay induced variation of synchronization bandwidth and frequency stability in a micromechanical oscillator. *Nonlinear Dyn.* **105**, 2981–2994 (2021).

29. M. C. Cross, Improving the frequency precision of oscillators by synchronization. *Phys. Rev. E* **85**, 046214 (2012).

30. D. K. Agrawal, A. A. Seshia, An analytical formulation for phase noise in MEMS oscillators. *IEEE Trans. Ultrason. Ferroelectr. Freq. Control* **61**, 1938–1952 (2014).

31. S. Walter, A. Nunnenkamp, C. Bruder, Quantum synchronization of a driven self-sustained oscillator. *Phys. Rev. Lett.* **112**, 094102 (2014).

32. F. Schmolke, E. Lutz, Measurement-induced quantum synchronization and multiplexing. *Phys. Rev. Lett.* **132**, 010402 (2024).

33. A. Roulet, C. Bruder, Quantum synchronization and entanglement generation. *Phys. Rev. Lett.* **121**, 063601 (2018).

34. H. Xiao, H. He, L. Zhang, T. Liu, Adaptive grid-synchronization based grid-forming control for voltage source converters. *IEEE Trans. Power Syst.* **39**, 4763–4766 (2023).

35. M. Rohden, A. Sorge, M. Timme, D. Witthaut, Self-organized synchronization in decentralized power grids. *Phys. Rev. Lett.* **109**, 064101 (2012).

36. A. Pikovsky, M. Rosenblum, J. Kurths, *Synchronization: A Universal Concept in Nonlinear Sciences* (Cambridge Univ. Press, 2010).

37. M. Tyloo, R. Delabays, P. Jacquod, Noise-induced desynchronization and stochastic escape from equilibrium in complex networks. *Phys. Rev. E* **99**, 062213 (2019).

38. D. S. Goldobin, A. Pikovsky, Synchronization and desynchronization of self-sustained oscillators by common noise. *Phys. Rev. E* **71**, 045201 (2005).

39. R. Adler, A study of locking phenomena in oscillators. *Proc. IRE* **34**, 351–357 (1946).

40. H.-C. Chang, X. Cao, U. K. Mishra, R. A. York, Phase noise in coupled oscillators: Theory and experiment. *IEEE Trans. Microw. Theory Tech.* **45**, 604–615 (1997).

41. N. Patin, L. Vido, E. Monmasson, J.-P. Louis, M. Gabsi, M. Lecrivain, Control of a hybrid excitation synchronous generator for aircraft applications. *IEEE Trans Ind Electron* **55**, 3772–3783 (2008).

42. A. Hajimiri, T. H. Lee, A general theory of phase noise in electrical oscillators. *IEEE J. Solid-State Circuits* **33**, 179–194 (1998).

43. T. H. Lee, A. Hajimiri, Oscillator phase noise: A tutorial. *IEEE J. Solid-State Circuits* **35**, 326–336 (2000).

44. J. Hindes, P. Jacquod, I. B. Schwartz, Network desynchronization by non-Gaussian fluctuations. *Phys. Rev. E* **100**, 052314 (2019).

45. C. Zhou, J. Kurths, Noise-induced phase synchronization and synchronization transitions in chaotic oscillators. *Phys. Rev. Lett.* **88**, 230602 (2002).

46. T. Manzaneque, M. K. Ghatkesar, F. Alijani, M. Xu, R. A. Norte, P. G. Steeneken, Resolution limits of resonant sensors. *Phys. Rev. Appl.* **19**, 054074 (2023).

47. Y. Xu, L. Wang, C. Wang, J. Ren, J. Lv, G. Shao, X. Wei, Frequency stabilization in a pseudo-linear micromechanical parametric oscillator. *Int. J. Mech. Sci.* **280**, 109610 (2024).

48. O. Shoshani, S. Strachan, D. Czaplewski, D. Lopez, S. W. Shaw, Extraordinary frequency stabilization by resonant nonlinear mode coupling. *Phys. Rev. Appl.* **22**, 054055 (2024).

49. X. Li, Positive-incentive noise. *IEEE Trans. Neural Netw. Learn. Syst.* **35**, 6 (2024).

50. Y. S. Teo, S. Shin, H. Kwon, S.-H. Lee, H. Jeong, Virtual distillation with noise dilution. *Phys. Rev. A* **107**, 022608 (2023).

51. J. Jhawar, R. G. Morris, U. R. Amith-Kumar, M. D. Raj, T. Rogers, H. Rajendran, V. Guttal, Noise-induced schooling of fish. *Nat. Phys.* **16**, 488–493 (2020).

52. C. A. Yates, R. Erban, C. Escudero, I. D. Couzin, C. Buhl, I. G. Kevrekidis, P. K. Maini, D. J. T. Sumpter, Inherent noise can facilitate coherence in collective swarm motion. *Proc. Natl. Acad. Sci. U.S.A.* **106**, 5464–5469 (2009).

53. C. C. Ioannou, V. Guttal, I. D. Couzin, Predatory fish select for coordinated collective motion in virtual prey. *Science* **337**, 1212–1215 (2012).

54. I. Kovacic, M. J. Brennan, *The Duffing Equation: Nonlinear Oscillators and Their Behaviour* (Wiley, 2011).

55. D. K. Agrawal, J. Woodhouse, A. A. Seshia, Observation of locked phase dynamics and enhanced frequency stability in synchronized micromechanical oscillators. *Phys. Rev. Lett.* **111**, 084101 (2013).

56. Y. Qiao, A. Elhady, M. Arabi, E. Abdel-Rahman, W. Zhang, Thermal noise-driven resonant sensors. *Microsyst. Nanoeng.* **10**, 90 (2024).

57. G. Marghoti, T. L. Prado, M. A. F. Sanjuán, S. R. Lopes, Beat frequency induced transitions in synchronization dynamics. *Commun. Nonlinear Sci. Numer. Simul.* **138**, 108243 (2024).

58. Z. Shi, D. Pu, Q. Lv, R. Huan, X. Wang, Z. Xiao, Z. Jiang, X. Wei, Enhancement of synchronization bandwidth in an arch beam. *J. Sound Vib.* **545**, 117415 (2023).

59. J. A. Barnes, A. R. Chi, L. S. Cutler, D. J. Healey, D. B. Leeson, T. E. McGunigal, J. A. Mullen, W. L. Smith, R. L. Sydnor, R. F. C. Vessot, G. M. R. Winkler, Characterization of frequency stability. *IEEE Trans. Instrum. Meas.* **20**, 105–120 (1971).

60. M. L. Deng, W. Q. Zhu, Stochastic averaging of MDOF quasi integrable Hamiltonian systems under wide-band random excitation. *J. Sound Vib.* **305**, 783–794 (2007).

61. Z. L. Huang, W. Q. Zhu, Stochastic averaging of quasi-integrable Hamiltonian systems under combined harmonic and white noise excitations. *Int. J. Nonlinear Mech.* **39**, 1421–1434 (2004).

62. R. L. Stratonovich, *Topics in the Theory of Random Noise* (Gordon and Breach, 1963).

63. R. Z. Khasminskii, A limit theorem for the solutions of differential equations with random right-hand sides. *Theory Probab. Appl.* **11**, 390–406 (1966).

64. A. H. Nayfeh, D. T. Mook, *Nonlinear Oscillations* (Wiley, 2008).

65. Z. Xiao, Z. Shi, X. Wang, X. Wei, R. Huan, Dual-jump amplification in an electric-thermal adjusted arch beam micro-resonator. *Sens. Actuators A Phys.* **365**, 114925 (2024).

66. M. Amabili, P. Balasubramanian, G. Ferrari, Nonlinear vibrations and damping of fractional viscoelastic rectangular plates. *Nonlinear Dyn.* **103**, 3581–3609 (2021).

67. M. Amabili, Nonlinear damping in nonlinear vibrations of rectangular plates: Derivation from viscoelasticity and experimental validation. *J. Mech. Phys. Solids* **118**, 275–292 (2018).
